# SPAAC Pulse-Chase: A Novel Click Chemistry-Based Method to Determine the Half-Life of Cellular Proteins

**DOI:** 10.3389/fcell.2021.722560

**Published:** 2021-09-07

**Authors:** Trevor M. Morey, Mohammad Ali Esmaeili, Martin L. Duennwald, R. Jane Rylett

**Affiliations:** ^1^Molecular Medicine Research Group, Robarts Research Institute, Western University, London, ON, Canada; ^2^Department of Physiology and Pharmacology, Western University, London, ON, Canada; ^3^Department of Anatomy and Cell Biology, Schulich School of Medicine & Dentistry, Western University, London, ON, Canada

**Keywords:** protein stability and degradation, protein half-life, pulse-chase analysis, click chemistry, SPAAC, mammalian cells, yeast

## Abstract

Assessing the stability and degradation of proteins is central to the study of cellular biological processes. Here, we describe a novel pulse-chase method to determine the half-life of cellular proteins that overcomes the limitations of other commonly used approaches. This method takes advantage of pulse-labeling of nascent proteins in living cells with the bioorthogonal amino acid L-azidohomoalanine (AHA) that is compatible with click chemistry-based modifications. We validate this method in both mammalian and yeast cells by assessing both over-expressed and endogenous proteins using various fluorescent and chemiluminescent click chemistry-compatible probes. Importantly, while cellular stress responses are induced to a limited extent following live-cell AHA pulse-labeling, we also show that this response does not result in changes in cell viability and growth. Moreover, this method is not compromised by the cytotoxicity evident in other commonly used protein half-life measurement methods and it does not require the use of radioactive amino acids. This new method thus presents a versatile, customizable, and valuable addition to the toolbox available to cell biologists to determine the stability of cellular proteins.

## Introduction

The stability and degradation of cellular proteins are critical parameters that influence and regulate most aspects of cellular physiology and many disease-associated processes. Targeted proteolysis of cellular proteins in a timely manner ensures that cells divide on cue, maintain their proper functions, or undergo apoptosis when appropriate (Varshavsky, 2008; Knecht et al., 2009). Consequently, dysregulated proteolysis of key proteins is associated with many human diseases (Hanna et al., 2019), for example the tumor suppressor p53 in cancer (Hengstermann et al., 2001; Mantovani et al., 2019), α-synuclein, β-amyloid, and other misfolding-prone proteins in neurodegenerative diseases (Baranello et al., 2015; Ciechanover and Kwon, 2015; Lehtonen et al., 2019), and folding-mutants of cystic fibrosis transmembrane conductance regulator in cystic fibrosis (Sharma et al., 2004). Thus, quantification of the rate of degradation of cellular proteins, i.e., determination of protein half-lives, is an essential tool when studying physiological and disease-related cellular processes and can provide insight into the stability, regulation, mechanisms of degradation, and function of cellular proteins.

To date, three major methods are commonly used to determine protein half-life, each of which has specific advantages but also severe limitations (Eldeeb et al., 2019). These include (1) treatment of cells with cycloheximide (CHX), a fast and effective, yet highly cytotoxic, inhibitor of protein synthesis (Schneider-Poetsch et al., 2010), (2) pulse-chase experiments that involve labeling of newly synthesized proteins in living cells with radioisotopic amino acids such as ^35^S-methionine (Takahashi and Ono, 2003; Esposito and Kinzy, 2014), and (3) expression of a protein of interest as a GFP (green fluorescent protein) fusion, where GFP is either photoactivatable (Zhang et al., 2007) or can be permanently photobleached (Eden et al., 2011). Radiolabeling of nascent cellular proteins is often considered the gold standard for pulse-chase analysis and involves minimal disturbance to normal cellular conditions. However, a significant disadvantage of this method is the use of potentially biohazardous radioisotopes, the requirements for special permits, and the use of specific protocols and equipment in a containment environment. Furthermore, radiolabeling can induce DNA and cellular damage, lead to cell cycle arrest, alter cell morphology, and induce apoptosis (Hu and Heikka, 2000; Hu et al., 2001). Alternatively, CHX can be used with standard laboratory equipment without the need for radioisotope precautions and is often preferred because of its simplicity. However, treatment of cells with CHX inhibits *de novo* protein synthesis and non-specifically affects a wide array of cellular processes, including kinase pathways and proteolytic machinery (Hanna et al., 2003; Dai et al., 2013). Thus, the use of CHX treatment is not suitable when studying proteins with long half-lives, and furthermore may affect the protein half-life of cellular proteins in unintended ways. Protocols involving either ^35^S-methionine pulse-chase labeling or CHX treatment require cell lysis, whereas GFP tags allow for monitoring protein half-life in living cells by microscopy. However, this latter method requires the heterologous expression of a GFP-tagged protein, and thus cannot be used to study endogenous proteins. Moreover, expression of GFP alone or as a fusion protein can induce proteome changes, alter kinase and ubiquitin signaling pathways, and cause cellular toxicity (Baens et al., 2006; Coumans et al., 2014; Ansari et al., 2016).

Considering these methodological shortcomings, we identified a need for a more reliable and less constrained method to determine the half-life of cellular proteins. A new approach designed originally to measure global changes in proteome dynamics involves the labeling of living cells with L-azidohomoalanine (AHA), a bioorthogonal methionine analog containing a reactive azide moiety that is selectively incorporated into newly synthesized proteins (Kiick et al., 2002; Dieterich et al., 2006). AHA-labeled proteins can then be reacted with an alkyne-containing molecule in a click chemistry reaction that allows for the isolation of AHA-labeled proteins for downstream proteomic analysis (McShane et al., 2016). A major advantage of AHA labeling over ^35^S-methionine labeling, CHX treatment, or the use of GFP fusion proteins is that AHA is non-toxic, non-radioactive, does not inhibit protein synthesis, and does not alter global protein ubiquitination or degradation (Dieterich et al., 2006). While AHA-labeling has been used previously both *in vitro* and *in vivo* to measure global changes in protein synthesis without affecting cellular viability (Dieterich et al., 2006, 2010; Baskin et al., 2007; Roche et al., 2009; McShane et al., 2016), this technique has not yet been applied to studying the stability and degradation of individual proteins-of-interest.

Here, we describe a novel pulse-chase procedure for the non-toxic and non-radioactive determination of cellular protein half-life using click chemistry that can monitor specific proteins-of-interest (Morey et al., 2016). This method is an adaptation of classical ^35^S-methionine pulse-chase labeling that utilizes copper-free strain-promoted alkyne-azide cycloaddition (SPAAC) reactions to conjugate a fluorescent or biotin cyclooctyne probe onto newly synthesized AHA-labeled proteins. Following immunoprecipitation of a protein-of-interest, half-life of an AHA-labeled protein can be monitored by standard SDS-PAGE and immunoblotting. We provide examples for the application of this method to measure the half-life of both endogenous and over-expressed proteins in mouse and human cell lines, and in yeast cells. We also include a proof-of-principle example that provides novel insights into cholinergic neurobiology (Morey et al., 2016). Additionally, we compare various commercially available fluorescent and biotin cyclooctyne probes for detection of AHA-labeled proteins.

Collectively, this method utilizes bioorthogonal click chemistry reactions in a manner that is compatible in a variety of cell systems and that allows user customization, thus establishing a versatile method with a wide applicability to molecular and cell biologists. To our knowledge, our study is the first to report on the use of click chemistry-based labeling to determine the protein half-life of specific proteins with high sensitivity. SPAAC pulse-chase thus presents a novel, reliable, and effective method to study protein stability in living cells.

## Materials and Methods

### Mammalian Cell Culture and Cell Lysis

Mouse cholinergic SN56 neural cells (gift from Dr. J. K. Blusztajn, Boston University) (Blusztajn et al., 1992) or human HEK293 or HeLa cells (ATCC) were grown as monolayers in DMEM supplemented with either 5% (SN56) or 10% FBS (HEK293 and HeLa; Invitrogen), and 1% Pen-Strep at 37°C with 5% CO_2_. Prior to experiments assessing the half-life of human 69-kDa choline acetyltransferase (ChAT) protein, cells were transiently transfected for 18–24 h at 37°C using either Lipofectamine 2000 or 3000 (Invitrogen) at ∼50% confluence with a plasmid encoding either wild-type or mutant proline-to-alanine P17A/P19A ChAT cDNA ligated to pcDNA3.1+ vector (Dobransky et al., 2000; Morey et al., 2016). Following treatments, cells were collected and lysed on ice in RIPA buffer (50 mM Tris–HCl; pH 8.0, 150 mM NaCl, 1% Triton X-100, 0.5% sodium deoxycholate, 0.1–0.5% SDS) supplemented with mammalian protease inhibitor cocktail (Sigma), phosphatase inhibitor cocktail (10 mM NaF, 1 mM Na_3_VO_4_, 20 mM Na_2_HPO_4_, 3 mM β-glycerolphosphate, 5 mM sodium pyrophosphate), 50 μM MG132, 10 mM N-ethylmaleimide (NEM; Calbiochem), and 800 U/ml DNase I (Invitrogen). Lysates were centrifuged for 10 min at 21,000 *g* at 4°C and protein concentrations were measured by BCA protein assay (Thermo). Aliquots of lysate supernatant were either used for immunoprecipitations or denatured in 1× Laemmli sample buffer (63 mM Tris–HCl; pH 6.8, 10% glycerol, 2% SDS, 0.005% bromophenol blue, 2.5% 2-mercaptoethanol) at 95°C for 10 min, then analyzed by SDS-PAGE and immunoblotting.

### SPAAC Pulse-Chase in Mammalian Cells

SN56 cells, transiently expressing either wild-type or mutant P17A/P19A-ChAT, or wild-type HEK293 cells were grown to ∼70% confluence prior to the start of SPAAC pulse-chase. To begin, cells were washed twice with methionine-free (Met-) DMEM (Invitrogen) to remove excess methionine then live-labeled (pulsed) at 37°C for 4 h with 50 μM Click-iT L-azidohomoalanine (AHA; Invitrogen) in Met- DMEM supplemented with 5–10% dialyzed FBS (Invitrogen). Control cells were grown for 4 h in methionine-containing (Met+) DMEM with 5–10% FBS. AHA-labeled cells were then washed twice with Met+ DMEM, and subsequently incubated in Met+ DMEM with 5–10% FBS for 2–24 h at 37°C (chase). Additionally, for determination of ChAT protein half-life during proteasome inhibition, SN56 cells were treated with 5 μM MG132 throughout both the 4 h pulse and 24 h chase period (i.e., 28 h). Cells were collected on ice either immediately following the pulse (control and 0 h) or following the chase periods (2, 4, 10, and 24 h), lysed in supplemented RIPA buffer (above) containing 0.5% SDS and 10 mM of freshly prepared iodoacetamide (Van Geel et al., 2012), then centrifuged for 10 min at 21,000 *g* at 4°C.

For fluorescent/biotin cyclooctyne labeling of AHA-labeled proteins from whole cell lysates, aliquots of cleared cell lysate were reacted with either 10 μM Click-iT TAMRA-DIBO (Invitrogen), 5 μM AFDye 488-DBCO (Click Chemistry Tools), or 10 μM Click-iT Biotin-DIBO (Invitrogen) for 1 h at 21°C followed by denaturation in 1× Laemmli sample buffer at 95°C for 10 min. For analysis, equal amounts of proteins (e.g., 25 μg) were resolved on SDS-PAGE gels and fluorescence was detected in-gel using a ChemiDoc MP system (Bio-Rad) at an excitation/emission of either 555/580 nm (TAMRA-DIBO) or 494/517 nm (488-DBCO). Alternatively, if reacted with Biotin-DIBO, protein samples were resolved on SDS-PAGE gels, transferred to PVDF membranes (Bio-Rad), then membranes were probed with Pierce High Sensitivity Streptavidin-HRP (Thermo) and Clarity Western ECL Substrate (Bio-Rad). As controls, AHA/cyclooctyne-labeled samples from whole cell lysates were transferred to PVDF membranes and standard immunoblotting was completed.

To determine the protein half-life of either ChAT or p53, anti-ChAT or anti-p53 IPs were prepared from cleared whole cell lysates of AHA-labeled cells as detailed below. Immunocaptured samples were washed twice with cold 0.5%-SDS RIPA buffer, twice with cold PBS to remove detergents, then subsequently reacted with strained cyclooctynes (10 μM TAMRA-DIBO, 5 μM 488-DBCO, or 10 μM Biotin-DIBO) in 1× PBS supplemented with mammalian protease inhibitors for 1 h at 21°C with gentle agitation. Samples were washed twice with cold 0.5%-SDS RIPA buffer and IP samples was eluted into 50 μl of 2× Laemmli sample buffer with 5% 2-mercaptoethanol at 85°C for 15 min with intermittent mixing. For analysis, equal volumes of immunoprecipitated AHA/cyclooctyne-labeled ChAT or p53 (e.g., 20 μl) were resolved on SDS-PAGE gels and either in-gel fluorescence (TAMRA-DIBO or 488-DBCO) or biotin (Biotin-DIBO) labeling was detected as above. Lastly, as controls, AHA/cyclooctyne-labeled IP samples were transferred to PVDF membranes and anti-ChAT or anti-p53 immunoblots were completed.

To calculate protein half-life, we assumed that the amount of AHA/cyclooctyne-labeled ChAT or p53, *P*(*t*), decays exponentially under first order kinetics according to the equation *P*(*t*) = P_o_e^–αt^, where P_o_ is the fluorescence (TAMRA-DIBO or 488-DBCO) or biotin-ECL (Biotin-DIBO) intensity at *t* = 0. The slope of decay (α) was calculated by plotting the intensity of immunoprecipitated AHA/cyclooctyne-labeled ChAT or p53, corrected for the levels of total immunoprecipitated ChAT or p53 as measured by parallel immunoblotting, on a semi-logarithmic scale and performing linear regression. ChAT and p53 protein half-life, T_1/2_, was calculated according to first order kinetics where *T*_1/2_ = ln(2)/α (Eden et al., 2011).

### Cycloheximide (CHX) Assay

SN56 cells were transfected and plated as for SPAAC pulse-chase to transiently express either wild-type or mutant P17A/P19A-ChAT, then treated with 100 μg/ml CHX for 2, 4, 6, or 8 h; control cells were treated with DMSO. Cells were collected and lysed on ice in supplemented 0.1%-SDS RIPA buffer, lysates were centrifuged for 10 min at 21,000 *g* at 4°C, then protein samples from cleared whole cell lysates were denatured in 1× Laemmli sample buffer at 95°C for 5 min. Protein samples were resolved on SDS-PAGE gels, transferred to PVDF membranes, then immunoblotting was completed. To determine the protein half-life of wild-type and P17A/P19A-ChAT, anti-ChAT immunoreactive bands were quantified by densitometry, plotted on a semi-logarithmic scale, and analyzed as for SPAAC pulse-chase by linear regression to determine a slope of decay.

### Serial Dilution Assay for Detection Sensitivity of Strained Cyclooctynes

HEK293 cells were live-labeled in culture for 4 h with 50 μM AHA in methionine-free DMEM then collected immediately without a methionine chase. Control unlabeled cells were incubated in Met+ DMEM. Cells were collected and lysed on ice in supplemented 0.1%-SDS RIPA buffer, lysates were centrifuged for 10 min at 21,000 *g* at 4°C, and aliquots of cleared whole cell lysate were reacted with either 10 μM TAMRA-DIBO, 5 μM 488-DBCO, or 10 μM Biotin-DIBO for 1 h at 21°C. Protein samples were denatured in 1× Laemmli sample buffer at 95°C for 10 min then were serially diluted 1:1 with 1× Laemmli sample buffer a total of six times until reaching a final dilution of 1:64. For analysis, 25 μg of total protein initially, then an equal volume from each serially diluted samples (1:64 = 0.39 μg total protein), were resolved on SDS-PAGE gels and either in-gel fluorescence (TAMRA-DIBO or 488-DBCO) or biotin (Biotin-DIBO) labeling was detected as above. Anti-actin immunoblots were completed as a loading control.

### Analysis of Cell Viability, Global Proteome Ubiquitination, Protein Solubility, and Heat Shock Response in Mammalian Cells Labeled With AHA

Mouse SN56 cells or human HEK293 or HeLa cells were live-labeled in culture for 4 h with 50 μM AHA and either collected immediately (0 h) or following 8 h of chase in Met+ DMEM. Control unlabeled cells were incubated in media with methionine (i.e., without AHA). To inhibit *de novo* protein synthesis SN56 cells were co-treated with 100 μg/ml CHX throughout both the 4 h AHA pulse and the 8 h chase periods (up to 12 h total); control unlabeled cells were treated with CHX for 4 h in Met+ DMEM, while control AHA-labeled cells were treated with DMSO. To induce apoptosis or protein misfolding, cells were treated in Met+ DMEM with either 200 nM staurosporine or 10 mM azetidine-2-caroxylic acid (AZC) for either 8 h or 24 h, respectively. Cells were lysed on ice in supplemented 0.1%-SDS RIPA buffer, lysates were centrifuged for 10 min at 21,000 *g* at 4°C, and aliquots of cleared whole cell lysate were reacted with 10 μM TAMRA-DIBO for 1 h at 21°C. Protein samples were then denatured in 1× Laemmli sample buffer at 95°C for 10 min, run on SDS-PAGE gels and AHA-labeled proteins were detected in-gel at an Ex/Em 555/580 nm. Subsequently, protein samples were transferred to PVDF membranes and immunoblotting was completed as indicated.

Alternatively, to determine if AHA may affect the overall solubility of cellular proteins, SN56 cells were live-labeled in culture for 4 h with 50 μM AHA and either collected immediately (0 h) or following 8 h of chase in Met+ DMEM as above. Additionally, as a positive control to induce protein misfolding cells were treated in Met+ DMEM with 10 mM AZC for 24 h. Cells were collected and lysed on ice in 0.1% Triton X-100 lysis buffer (50 mM Tris–HCl; pH 8.0, 150 mM NaCl, 0.1% Triton X-100) supplemented with protease/phosphatase inhibitors, 50 μM MG132, and 10 mM NEM. Lysates were centrifuged for 15 min at 15,000 *g* at 4°C and aliquots of Triton-soluble supernatant were prepared for immunoblotting by denaturing in 1× Laemmli sample buffer at 95°C for 10 min. To prepare Triton-insoluble proteins for immunoblotting the Triton-insoluble pellets were washed once with ice-cold PBS, then denatured in an equal volume of 2× Laemmli sample buffer with 5% 2-mercaptoethanol at 85°C for 15 min prior to separation on SDS-PAGE gels and immunoblotting.

### Immunoprecipitation (IP)

For both anti-ChAT and anti-p53 IPs, cells were grown and treated on either 60 or 100 mm culture dishes to ∼90% confluence prior to collection. Cells were lysed in supplemented RIPA buffer (above) containing 0.5% SDS, then lysates were centrifuged for 10 min at 21,000 *g* at 4°C. Aliquots of cell lysate supernatants containing 1 mg protein were diluted to a final volume of 1 ml (1 mg/ml final) in supplemented RIPA buffer, then IP samples were incubated at 4°C for 18 h with either 2.5 μg of anti-ChAT primary antibody (CTab) (Dobransky et al., 2000) or with 2 μg of anti-p53 primary antibody (DO-1; Santa Cruz) per mg protein. Immune complexes were captured onto 50 μl of protein-G Dynabeads (Invitrogen) for 1 h at 4°C, then washed with cold RIPA buffer and used for SPAAC pulse-chase as detailed above.

### SDS-PAGE and Immunoblotting

Denatured protein samples from whole cell lysates and IPs were resolved on 7.5, 10, or 12% SDS-PAGE gels, then transferred to PVDF membranes by semi-dry electroblotting. For immunoblotting, membranes were blocked for 1 h at 21°C in 5% non-fat milk powder in PBS (137 mM NaCl, 2.7 mM KCl, 10 mM Na_2_HPO_4_, 1.8 mM KH_2_PO_4_; pH 7.4) containing 0.15% Triton X-100 (PBST) followed by incubation overnight at 4°C with primary antibody. Probed membranes were washed with PBST, then primary antibodies were detected using 1:10,000 peroxidase-coupled secondary antibodies (Jackson ImmunoResearch) and Clarity Western ECL Substrate on a ChemiDoc MP system. The following primary antibodies were used: 1:1000 ChAT (CTab) (Dobransky et al., 2000), 1:10,000 β-actin (Sigma), 1:1000 ubiquitin (Santa Cruz), 1:1000 p53 (DO-1; Santa Cruz), and 1:500 vinculin (Santa Cruz). For detection of biotinylated proteins following SDS-PAGE, membranes were blocked overnight at 4°C in PBST with 5% BSA, then incubated for 1 h at 21°C with 1:20,000 Pierce High Sensitivity Streptavidin-HRP, washed with PBST, and imaged using Clarity ECL as above.

### Yeast Strains and Media

Yeast strains BY 4741 and BY Δ *pdr5* were obtained from the *Saccharomyces* Genome Deletion Project. Standard yeast media were used^1^. Yeast transformations were performed according to a standard PEG/lithium acetate protocol as described before (Kawai et al., 2010). For induction of gene expression driven by the GAL1 promotor, 2% galactose was used instead of glucose as a carbon source for liquid media.

### SPAAC Pulse-Chase in Yeast

Liquid cultures of yeast cells (Δ *pdr5*) transformed with human ChAT expression plasmid were grown in non-inducing selective media (SD-leu) at 30°C for 24 h. Cells were pelleted, washed twice with sterile H_2_O, and cells were resuspended in 2% galactose containing media lacking methionine. SPAAC pulse-chase was initiated by the addition of 50 μM AHA and incubation of cells for 24 h at 30°C. Control cells were incubated for 24 h in methionine-containing media. Cells were pelleted and AHA labeling was subsequently terminated by first washing cells with sterile H_2_O then incubating them at 30°C for 2, 4, or 8 h in methionine-containing non-inducing SD-leu media. To inhibit the proteasome or lysosome, cells were co-treated with either 50 μM MG132 or 10 μM Bafilomycin A, respectively, throughout both the 24 h AHA-pulse and 8 h chase periods. Cells were collected, washed twice on ice with sterile H_2_O, and lysed in cold lysis buffer (50 mM HEPES; pH 7.5, 150 mM NaCl, 5 mM EDTA, 1% Triton X-100) supplemented with SIGMAFAST Protease inhibitor (Sigma), and 50 mM NEM. Sterile glass beads were added, cells were vortexed, and lysates were centrifuged for 10 min at 21,000 *g* at 4°C.

Aliquots of cleared protein lysates were reacted with 10 μM TAMRA-DIBO for 4 h at 21°C followed by denaturation in 1× Laemmli sample buffer at 95°C for 10 min. For analysis, equal amounts of proteins from each experimental sample were resolved on SDS-PAGE gels and fluorescence was detected in-gel using a ChemiDoc MP system at an excitation/emission of 555/580 nm. To determine ChAT protein half-life in yeast, anti-ChAT IPs were prepared from cleared whole cell lysates of AHA-labeled cells as detailed above. Immunocaptured samples were washed and subsequently reacted with 10 μM TAMRA-DIBO in 1× PBS for 1 h at 21°C with gentle agitation. IP samples were washed then eluted into 50 μl of 2× Laemmli sample buffer at 85°C for 15 min with intermittent mixing. For analysis, equal volumes of immunoprecipitated AHA/TAMRA-labeled ChAT were resolved on SDS-PAGE gels and in-gel fluorescence was detected as described above. As controls, AHA/TAMRA-labeled proteins from either whole cell lysates or ChAT IPs were transferred to PVDF membranes and standard immunoblotting was completed. ChAT protein half-life in yeast was calculated as detailed above.

### Analysis of Cell Growth and the Heat Shock Response in Yeast Cells Labeled With AHA

Cell growth was assessed by liquid culture as previously described (Duennwald, 2013). Briefly, liquid cell cultures were diluted to OD_600_ 0.15 and incubated at 30°C. OD_600_ was measured every 15 min using a Bioscreen C plate reader (Growth Curves USA) for 24 h. Growth curves were generated and the statistical significance was determined using a two-tailed student *t*-test and GraphPad Prism. To measure the effect of AHA labeling on induction of the heat shock response in yeast, a fluorescent reporter system whereby expression of heterologous GFP is driven by binding of heat shock factor 1 (Hsf1) to a synthetic promoter containing four adjacent heat shock elements (HSE) was used (Brandman et al., 2012). The cells were grown in inducing selective media (SD-leu) at 24°C for 24 h and/or 42°C for 1 h. Cells were pelleted, washed twice with sterile H_2_O, and cells were resuspended in media containing of 50 μM AHA and incubating cells for another 4 h at 24°C and/or 42°C. Cells were pelleted and AHA labeling was subsequently terminated by first washing cells with sterile H_2_O then cells were collected for imaging using Cytation 5 Cell Imaging Multi-Mode Reader (BioTek), and/or lysed on ice in 0.1% Triton X-100 lysis buffer (50 mM Tris–HCl; pH 8.0, 150 mM NaCl, 0.1% Triton X-100) supplemented with protease/phosphatase inhibitors. Lysates were centrifuged for 15 min at 15,000 *g* at 4°C, and immunoblotting. Immunoblotting was completed as described above. The following primary antibodies were used: 1:1000 Hsp104, Hsp42, and Hsp26 (gifts from J. Buchner), 1:1000 histone H3 (LSBio), and 1:1000 HSP70 (Santa Cruz).

### Statistical Analysis

Steady-state proteins levels from immunoblots were measured by densitometry of immunoreactive bands using ImageLab 5.0 software (Bio-Rad), normalized to either β-actin, PGK1, or histone H3 and graphed as mean ± SEM from individual independent replicate experiments (n). Statistical analysis for experiments was completed by one-way ANOVA with either Dunnett’s or Tukey’s *post hoc* test using GraphPad Prism software. Statistical significance was set at *p* ≤ 0.05.

## Results

### Overview of a Novel SPAAC Pulse-Chase Method

**FIGURE 1:**
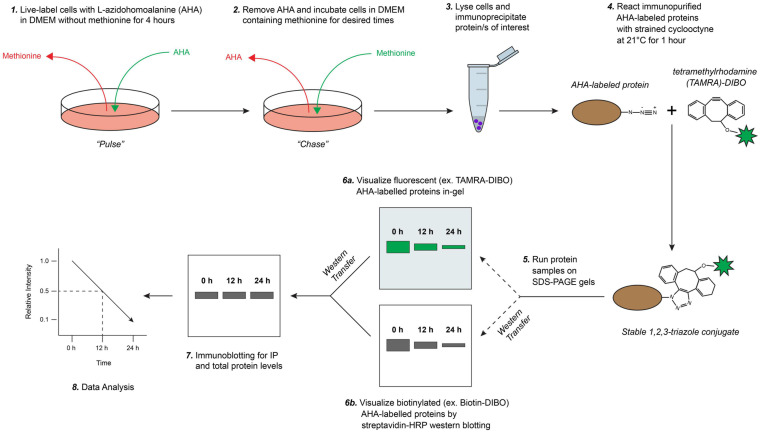
Schematic overview of biorthogonal strain-promoted alkyne-azide cycloaddition (SPAAC) pulse-chase for fluorescent/chemiluminescent determination of protein half-life. **(1)** Briefly, cultured cells are live-labeled (pulsed) with L-azidohomoalanine (AHA), a bioorthogonal methionine analog that contains a reactive azide moiety, under methionine-free conditions. **(2)** AHA-containing media is removed and labeled cells are first washed then chased with media containing methionine for desired times (e.g., up to 24 h). **(3)** Cells are collected, lysed, and protein/s of interest are immunoprecipitated. **(4)** Immunopurified AHA-labeled proteins are reacted with a strained cyclooctyne (e.g., 4-dibenzocyclooctynol; DIBO) that is modified with either a fluorescent (e.g., tetramethylrhodamine; TAMRA) or biotin probe to form stable triazole conjugates. **(5)** AHA/cyclooctyne-labeled proteins are resolved on SDS-PAGE gels and either **(6a)** fluorescence is detected directly in-gel (e.g., labeled with TAMRA-DIBO) or, **(6b)** if labeled with a biotin-cyclooctyne probe, proteins are then transferred onto PVDF membranes and detected by chemiluminescence using a HRP-conjugated streptavidin. **(7)** Lastly, immunoblotting is completed to measure steady-state and immunoprecipitated protein levels from AHA/cyclooctyne-labeled protein samples, and **(8)** subsequently for downstream data analysis. Adapted with permission from Morey et al. (2016).

We developed a novel pulse-chase method to determine the half-life of cellular proteins based on SPAAC click chemistry reactions using non-radioactive labeling and detection reagents (Figure 1). Briefly, in this method newly synthesized proteins are first live-labeled (pulsed) with AHA, a biorthogonal methionine analog that contains a reactive azide moiety, in cultured cells under methionine-free conditions and then chased with excess methionine. Cells are collected at specified times in chase media, lysed, and a protein-of-interest is immunoprecipitated (IP) according to established laboratory protocols. AHA-labeled proteins are then reacted via 1,3-dipolar cycloaddition to form stable 1,2,3-triazole conjugates with a strained cyclooctyne, such as 4-dibenzocyclooctynol (DIBO) or dibenzocyclooctyne (DBCO), that are modified with either a fluorescent (e.g., tetramethylrhodamine (TAMRA) or Alexa Fluor 488) or a biotin probe (Sanders et al., 2011; Kim et al., 2012; Dommerholt et al., 2016). AHA/cyclooctyne-labeled proteins are then resolved on SDS-PAGE gels and protein half-life can be determined either in-gel (fluorescent) or following transfer to a PVDF membrane (biotin). Lastly, for analysis standard immunoblotting is performed on AHA/cyclooctyne-labeled protein samples, thus eliminating the need to prepare both AHA-labeled and non-labeled samples and reducing potential sources of error.

### Determination of ChAT Protein Half-Life by SPAAC Pulse-Chase

**FIGURE 2:**
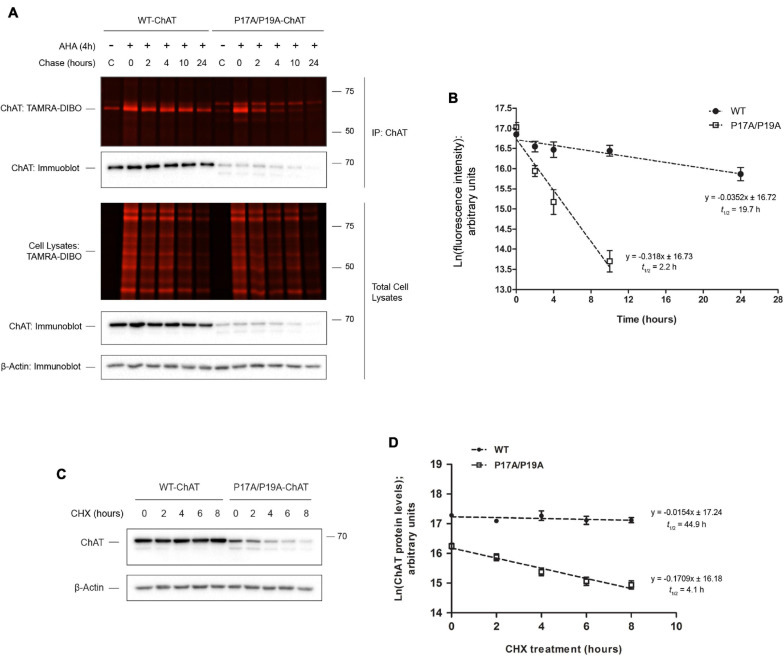
SPAAC pulse-chase reveals that ChAT protein half-life is reduced by mutation of an N-terminal proline-rich motif. **(A)** Fluorescence detection of immunoprecipitated (IP) AHA/TAMRA-labeled wild-type (WT) and P17A/P19A-ChAT from transiently transfected SN56 cells following SPAAC pulse-chase (4 h AHA pulse, 0–24 h methionine chase) with the strained cyclooctyne TAMRA-DIBO. Control unlabeled cells were incubated in media with methionine (i.e., without AHA). Fluorescent AHA/TAMRA-labeled proteins from either whole cell lysates or anti-ChAT IPs were detected in resolved SDS-PAGE gels at an Ex/Em of 555/580 nm. Anti-ChAT and anti-actin immunoblots were completed on AHA/TAMRA-labeled protein samples for downstream data analysis and as loading controls. **(B)** Protein half-life of P17A/P17A-ChAT (2.2 h) is reduced compared to wild-type ChAT (19.7 h). ChAT fluorescence intensities from **(A)** were plotted on a semi-logarithmic scale and linear regression was completed to determine ChAT protein half-life. The slopes of decay for wild-type and P17A/P19A-ChAT were significantly different: *F*(1,41) = 110.043, *p* ≤ 0.0001, *n* = 5. A 24 h time point was not included for the quantification of P17A/P19A-ChAT half-life as the fluorescence intensity was indistinguishable from background. **(C)** SN56 cells, transiently expressing either wild-type or P17A/P19A-ChAT, were treated with 100 μg/ml cycloheximide (CHX) for the time points indicated and anti-ChAT immunoblots were completed. Anti-actin immunoblots were completed as a loading control. **(D)** Protein half-life of P17A/P17A-ChAT (4.1 h) is reduced as compared to wild-type ChAT (44.9 h). Steady-state ChAT protein levels from **(C)** were plotted on a semi-logarithmic scale and linear regression completed to determine wild-type ChAT protein half-life. The slopes of decay for wild-type and P17A/P19A-ChAT were significantly different: *F*(1,46) = 36.802, *p* ≤ 0.0001, *n* = 5. Importantly, both methods **(B,D)** demonstrated that the protein half-life of P17A/P19A-ChAT is ∼10% that of wild-type ChAT. Adapted with permission from Morey et al. (2016).

We used this new method initially to address a previously unresolved question related to the cellular protein half-life of choline acetyltransferase (ChAT), the enzyme that catalyzes synthesis of the neurotransmitter acetylcholine (ACh) (Oda, 1999; Abreu-Villaca et al., 2011). ChAT mutations are linked to congenital myasthenic syndrome (CMS), a rare neuromuscular disorder (Engel et al., 2015). The CMS-related ChAT mutation V18M reduces enzyme activity and cellular protein levels (Shen et al., 2011) and is located within a highly conserved proline-rich motif at residues ^14^PKLPVPP^20^ that shares homology with SH3-binding motifs. Work from our laboratory found that disruption of this proline-rich motif reduces ChAT protein levels and cellular enzymatic activity of mutant P17A/P19A-ChAT and V18M-ChAT in mouse cholinergic SN56 cells (Morey et al., 2016). This reduction in cellular protein levels of mutant ChAT appeared to be due to enhanced ubiquitination, and thus we aimed to determine if the half-life of mutant ChAT protein is also reduced (Figure 2). Using this new SPAAC pulse-chase method in ChAT-expressing SN56 cells (Morey et al., 2016), we initially tested the method by first detecting the progressive global loss of fluorescent AHA/TAMRA-labeled proteins from whole cell lysates during the 0–24 h chase period in the absence of changes in total protein levels as measured in parallel by anti-actin immunoblotting (Figure 2A). Additionally, following anti-ChAT IPs and reaction of immunocaptured ChAT protein with the strained cyclooctyne TAMRA-DIBO, we observed progressive loss of fluorescent AHA/TAMRA-labeled ChAT protein during the 0–24 h chase period. As anticipated, the decay of P17A/P19A-ChAT protein appeared more rapid than that of wild-type ChAT. By quantifying the fluorescence intensities of AHA/TAMRA-labeled ChAT, we determined that the protein half-life of mutant P17A/P19A-ChAT (2.2 h) is significantly reduced by ∼10-fold compared to wild-type ChAT [19.7 h; *F*(1,41) = 110.043, *p* ≤ 0.0001; Figure 2B].

To compare the results generated using our new protocol to an established method, we performed a CHX assay on SN56 cells transiently expressing wild-type or P17A/P19A-ChAT. Unfortunately, we could only treat SN56 cells for 8 h due to the potent toxicity of CHX on these cells. Thus, by anti-ChAT immunoblotting of lysates from ChAT-expressing SN56 cells treated with 100 μg/ml CHX for 2–8 h (Figure 2C), we determined that the cellular half-life of P17A/P19A-ChAT (4 h) is again ∼10-fold shorter than that of wild-type ChAT [45 h; *F*(1,46) = 36.802, *p* ≤ 0.0001; Figure 2D]. Importantly, both methods revealed that the relative protein half-life of P17A/P19A-ChAT is ∼10% that of wild-type ChAT.

**FIGURE 3:**
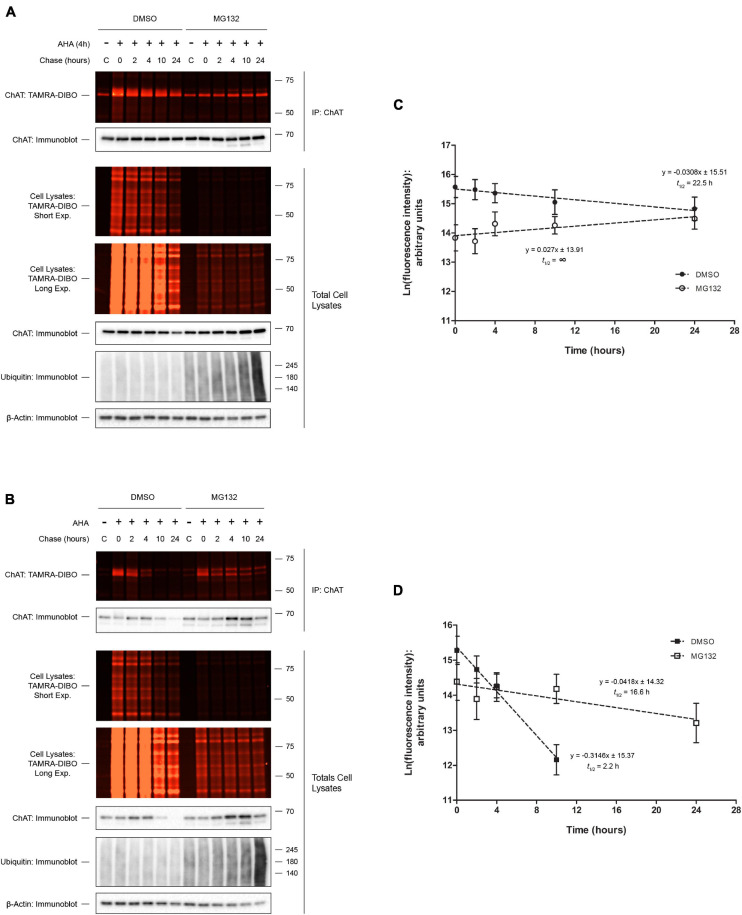
Proteasome inhibition increases ChAT protein half-life. Fluorescence detection of immunoprecipitated (IP) AHA/TAMRA-labeled wild-type (WT; **A**) or P17A/P19A-ChAT **(B)** from transiently transfected SN56 cells following SPAAC pulse-chase (4 h AHA pulse, 0–24 h methionine chase) with the strained cyclooctyne TAMRA-DIBO. Cells were treated with either DMSO-control or 5 μM MG132 throughout both the 4 h AHA pulse and the 24 h chase periods (up to 28 h total). Control unlabeled cells were incubated in media with methionine (i.e., without AHA). Fluorescent AHA/TAMRA-labeled proteins from either whole cell lysates or anti-ChAT IPs were detected in resolved SDS-PAGE gels at an Ex/Em of 555/580 nm. Anti-ChAT and anti-actin immunoblots were completed on AHA/TAMRA-labeled protein samples for downstream data analysis and as loading controls. **(C)** Proteasome inhibition by MG132 treatment increased the protein half-life of wild-type ChAT (no protein decay) as compared to DMSO-treated control cells (22.5 h). Wild-type ChAT fluorescence intensities from **(A)** were plotted on a semi-logarithmic scale and linear regression was completed to determine wild-type ChAT protein half-life. The slopes of decay for DMSO- and MG132-treated cells were significantly different from each other: *F*(1,46) = 4.79241, *p* ≤ 0.05, *n* = 5. **(D)** Proteasome inhibition by MG132 treatment increased the protein half-life of mutant P17A/P19A-ChAT (16.6 h) as compared to DMSO-control (2.2 h). ChAT fluorescence intensities from **(B)** were plotted on a semi-logarithmic scale and linear regression was completed to determine P17A/P19A-ChAT protein half-life. The slopes of decay for DMSO- and MG132-treated cells were significantly different from each other: *F*(1,41) = 18.9864, *p* ≤ 0.0001, *n* = 5. A 24 h time point was not included for the quantification of P17A/P19A-ChAT half-life in DMSO-treated cells as the fluorescence intensity was indistinguishable from background.

Our previously published results showed that inhibition of proteasome function by MG132 treatment, but not lysosomal function by chloroquine treatment, resulted in increased steady-state protein levels of both wild-type and P17A/P19A-ChAT (Morey et al., 2016, 2017). Furthermore, we have shown that MG132 treatment resulted in stabilization of ubiquitinated wild-type and P17A/P19A-ChAT, suggesting that ChAT protein degradation is regulated through the proteasome. Therefore, we tested whether proteasome inhibition by MG132 treatment correlates with an increase in ChAT protein half-life (Figure 3). Thus, we performed SPAAC pulse-chase on SN56 cells transiently expressing either wild-type (Figure 3A) or mutant P17A/P19A-ChAT (Figure 3B) that were treated with 5 μM MG132 throughout both the 4 h pulse and 24 h chase periods. ChAT protein was recovered from AHA-labeled SN56 cells by IP and subsequently fluorescence intensities of AHA/TAMRA-label ChAT were quantified. We observed that MG132 treatment prevented the decay of wild-type ChAT protein when compared to DMSO-treated control cells [half-life = 22.5 h; *F*(1,46) = 4.79241, *p* ≤ 0.05; Figure 3C]. Additionally, the half-life of P17A/P19A-ChAT protein was also increased during MG132 treatment (16.8 h) when compared to DMSO-control [2.2 h; *F*(1,41) = 18.9864, *p* ≤ 0.0001; Figure 3D]. Overall, these initial experiments provided novel insight into the proteolytic regulation of human ChAT protein and demonstrated that SPAAC pulse-chase is a valid and effective method to determine cellular protein half-life that is also compatible with inhibitors of protein degradation.

### Analysis of Cell Viability, Global Proteome Ubiquitination, Protein Solubility, and Heat Shock Response in Cells Labeled With AHA

Labeling of cells with AHA has been widely used in the past and shown to be non-toxic, does not inhibit protein synthesis, and does not alter global protein ubiquitination or degradation (Kiick et al., 2002; Dieterich et al., 2006, 2010; Baskin et al., 2007; Roche et al., 2009; McShane et al., 2016). To confirm these findings in the context of our experiments, we incubated SN56 cells for 4 h with 5 μM AHA in Met- media (pulsed), then performed a chase in Met+ media for 8 h. As a control to prevent labeling of newly synthesized proteins with AHA, we co-treated cells with 100 μg/ml CHX as shown in previous studies (Dieterich et al., 2006; Supplementary Figure 1A). Importantly, incubation of cells with AHA alone failed to either induce apoptosis or alter global ubiquitination, whereas treatment with CHX throughout both the 4 h AHA pulse and the 8 h chase periods significantly induced apoptosis (Supplementary Figure 1B; *p* ≤ 0.05) and led to a depletion in global protein ubiquitination (Supplementary Figure 1C; *p* ≤ 0.01) when compared to DMSO-treated control cells.

While used previously both *in vitro* and *in vivo* without compromising cell or animal viability (Kiick et al., 2002; Dieterich et al., 2006, 2010; Baskin et al., 2007; Roche et al., 2009; Hinz et al., 2012; Calve et al., 2016; McShane et al., 2016), incorporation of AHA into nascent proteins may induce changes to protein folding, leading to induction of the heat shock response (HSR). This response can be assessed by immunoblot for elevated levels in the heat shock proteins HSP70, HSP90, and HSC70. When assayed in SN56 cells, we observed increased steady state levels of HSP70 (Supplementary Figure 1A) and HSP90 and HSC70 proteins (Supplementary Figures 1D,E; *p* ≤ 0.05) in AHA-labeled cells after 8 h of chase when compared to untreated cells. As a positive control, treatment of cells with 10 mM azetidine-2-caroxylic acid (AZC), a proline analog that induces protein misfolding (Weids et al., 2016), for 24 h also promoted the synthesis of these HSPs (Lee and Seo, 2002). Of note, HSP levels were significantly greater in AZC-treated cells compared to AHA-treated cells (Supplementary Figures 1D–F, respectively; *p* ≤ 0.001).

Terminally misfolded proteins are often targeted for degradation through enhanced ubiquitination and proteasomal and/or lysosomal degradation (Amm et al., 2013). Thus, to determine whether the observed elevation in HSPs correlated with AHA-induced misfolding and subsequent insolublization of proteins, we fractionated whole cell lysates from AHA-labeled SN56 cells into Triton-soluble and -insoluble fractions. Importantly, following anti-ubiquitin immunoblotting (Supplementary Figure 2A), we did not observe detectable changes in the abundance of total ubiquitinated proteins in either the Triton-soluble (Supplementary Figure 2B) or Triton–insoluble fraction (Supplementary Figure 2C) from AHA-labeled cells when compared to unlabeled cells. As a positive control, treatment of cells with 10 mM AZC for 24 h led to the accumulation of total ubiquitinated proteins in both the Triton-soluble (Supplementary Figure 2B; *p* ≤ 0.001) and Triton–insoluble fraction (Supplementary Figure 2C; *p* ≤ 0.001) when compared to control and AHA-treated cells.

We also measured the effect of AHA labeling on cellular toxicity and induction of the HSR in HEK293 and HeLa cells, two commonly used human cells lines. Similar to SN56 cells, we observed by immunoblotting (Supplementary Figure 3A) that incubation with 50 μM AHA failed to induce apoptosis or lead to changes in global protein ubiquitination in either HEK293 (Supplementary Figures 3B,D) or HeLa cells (Supplementary Figures 3C,E). Interestingly, in HEK293 cells AHA labeling had no effect on the steady-state levels of HSP90, HSP70, or HSC70 (Supplementary Figures 3F,H,J, respectively), whereas in HeLa cells we observed an increase in HSP90 and HSP70 protein in AHA-labeled cells as compared to untreated cells (Supplementary Figures 3G,I; *p* ≤ 0.05). HSC70 protein levels were unchanged in HeLa cells following AHA labeling (Supplementary Figure 3K).

Taken together, these data suggest that AHA does not negatively affect cell viability or cellular protein degradation in the three mammalian cell lines tested here. While we do report that AHA treatment can induce the HSR, this was not universally observed and, importantly, does not correlate with either gross protein insolublization or cellular toxicity.

### Comparison of Commercially Available Strained Cyclooctyne Probes

**FIGURE 4:**
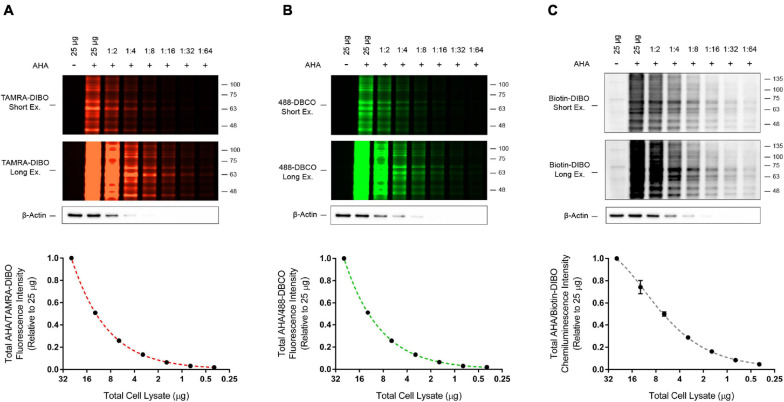
Detection sensitivity comparison for three different strained cyclooctyne probes. HEK293 cells were live-labeled in culture for 4 h with 50 μM AHA then collected immediately without a methionine chase. Control unlabeled cells were incubated in media with methionine (i.e., without AHA). Cells were lysed and AHA-labeled protein samples from whole cell lysates were reacted with the strained cyclooctynes **(A)** TAMRA-DIBO, **(B)** 488-DBCO, or **(C)** Biotin-DIBO. AHA/cyclooctyne-labeled proteins were denatured in 1× Laemmli sample buffer, then serially diluted 1:1 with 1× Laemmli sample buffer a total of six times until reaching a final dilution of 1:64. Protein samples were run on SDS-PAGE gels and AHA-labeled proteins were detected in-gel at an Ex/Em of either 555/580 nm (**A**; TAMRA-DIBO) or 494/517 nm (**B**; 488-DBCO). Alternatively, AHA-labeled proteins reacted with Biotin-DIBO **(C)** were resolved on SDS-PAGE gels, then transferred to PVDF membranes and detected by chemiluminescence using HRP-conjugated streptavidin. Anti-actin immunoblots were completed as a loading control. Overall, similar detection sensitivity was observed between AHA-labeled protein samples reacted with TAMRA-DIBO, 488-DBCO, or Biotin-DIBO (*n* = 4).

An advantage we propose for SPAAC pulse-chase is that various strained cyclooctyne reagents with unique probes can be conjugated to AHA-labeled proteins, thus increasing the versatility of this method. In addition to the TAMRA-DIBO label used in the experiments described above, we also prepared whole cell lysates from AHA-labeled HEK293 cells and compared conjugation of two other commercially available strained cyclooctynes, 488-DBCO and Biotin-DIBO, to that of TAMRA-DIBO. To test the sensitivity of these three strained cyclooctynes, we made serial dilutions of protein samples containing AHA/cyclooctyne-labeled proteins and ran them on SDS-PAGE gels. Signal intensities were then measured either in-gel when using fluorescent TAMRA-DIBO (Figure 4A) or 488-DBCO (Figure 4B), or the sensitivity of Biotin-DIBO (Figure 4C) was determined using HRP-conjugated streptavidin following transfer to PVDF membrane. Overall, we successfully conjugated these three different strained cyclooctyne probes to AHA-labeled proteins and observed little (Biotin-DIBO) or no (TAMRA-DIBO and 488-DBCO) background signal in protein samples from unlabeled cells. Furthermore, after lysates were serially diluted, we observed a similar signal sensitivity when using either TAMRA-DIBO, 488-DBCO, or Biotin-DIBO, and obtained robust fluorescent or chemiluminescent signals with as little as 0.39 μg of total protein (1:64 dilution from an initial 25 μg of protein).

### Determination of Endogenous p53 Protein Half-Life by SPAAC Pulse-Chase

**FIGURE 5:**
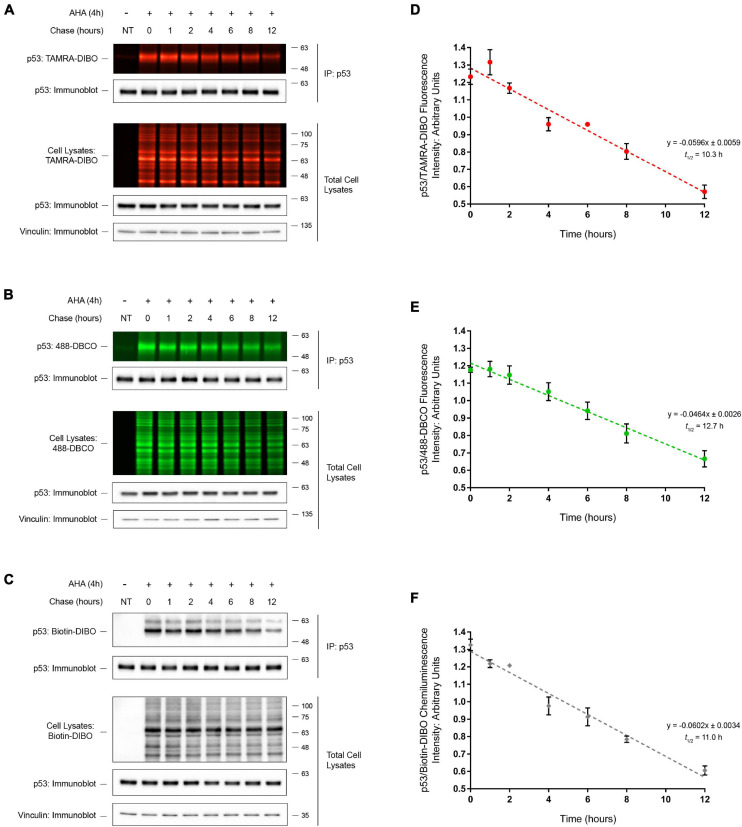
Half-life determination of p53 by SPAAC pulse-chase. Detection of immunoprecipitated (IP) endogenous AHA-labeled p53 from HEK293 cells following SPAAC pulse-chase (4 h AHA pulse, 0–12 h methionine chase) with the strained cyclooctynes TAMRA-DIBO **(A)**, 488-DBCO **(B)**, or Biotin-DIBO **(C)**. Control unlabeled cells were incubated in media with methionine (i.e., without AHA). AHA/cyclooctyne-labeled proteins from either whole cell lysates or anti-p53 IPs were resolved on SDS-PAGE gels and AHA-labeled proteins were detected in-gel at an Ex/Em of either 555/580 nm (**A**; TAMRA-DIBO) or 494/517 nm (**B**; 488-DBCO). Alternatively, AHA-labeled proteins reacted with Biotin-DIBO **(C)** were resolved on SDS-PAGE gels, then transferred to PVDF membranes and detected by chemiluminescence using a HRP-conjugated streptavidin. Anti-p53 and anti-vinculin immunoblots were completed on AHA/cyclooctyne-labeled protein samples for downstream data analysis and as loading controls. The protein half-life of AHA-labeled p53 when reacted with three different strained cyclooctynes was determined to be 10.3 h (**D**; TAMRA-DIBO), 12.7 h (**E**; 488-DBCO), or 11.0 h (**F**; Biotin-DIBO). Fluorescent or chemiluminescent intensities from immunoprecipitated p53 were plotted on a linear scale and linear regression analysis was completed to determine p53 protein half-life (*n* = 5).

We next tested whether this method could be used to determine the half-life of an endogenous protein-of-interest. To this end, we performed SPAAC pulse-chase analysis of the tumor suppressor protein p53, a protein that is essential for maintaining genomic stability and that is commonly mutated in many human cancers (Mantovani et al., 2019). Following AHA-labeling of HEK293 cells and a 0–12 h chase period, AHA-labeled proteins were reacted with the strained cyclooctynes TAMRA-DIBO (Figure 5A), 488-DBCO (Figure 5B), or Biotin-DIBO (Figure 5C), and protein samples were resolved by SDS-PAGE. As anticipated, we detected the global progressive loss of fluorescent (TAMRA-DIBO or 488-DBCO) and biotin-DIBO-labeled proteins from whole cell lysates during the 0–12 h chase period in the absence of changes in total protein levels as measured in parallel by anti-actin immunoblotting. Additionally, following IP of endogenous p53 we were able to observe the progressive loss of AHA/cyclooctyne-labeled p53 protein during the 0–12 h chase period using all three of the strained cyclooctyne probes tested. The half-life of AHA-labeled endogenous p53 protein was determined to be 10.3 h (Figure 5D; TAMRA-DIBO), 12.7 h (Figure 5E; 488-DBCO), or 11.0 h (Figure 5F; Biotin-DIBO), respectively, thus demonstrating that these different cyclooctyne probes produce similar results that are comparable to previously published data (Lukashchuk and Vousden, 2007; Dai et al., 2013). Together, these data suggest that not only can SPAAC pulse-chase be used to determine the half-life of endogenous proteins, but also that various commercially available strained cyclooctyne probes can be used interchangeably without dramatically altering the resulting protein half-lives.

### SPAAC Pulse-Chase in Yeast

We next sought to demonstrate the broader applicability of our method by expanding it beyond mammalian cells into the model organism yeast (*Saccharomyces cerevisiae*). One advantage to using yeast is that, unlike constitutive promoters used typically in many mammalian systems, a multitude of selectively inducible promoters exists for transgene expression (Weinhandl et al., 2014). Thus, using human ChAT as a protein of interest for SPAAC pulse-chase in yeast, we generated expression vectors for either wild-type 69-kDa human ChAT or a yellow fluorescent protein (YFP)-tagged ChAT protein under the control of a galactose-inducible promoter. As yeast do not naturally express a ChAT ortholog, we first assessed whether the expression of human ChAT has an effect on the growth of yeast cultures under normal conditions. Spotting assays on agar plates (Supplementary Figure 4A) and growth curves in liquid media (Supplementary Figure 4B) showed no growth defect associated with the heterologous expression of human ChAT in yeast. Furthermore, in agreement with work in mammalian cells (Resendes et al., 1999), YFP-tagged ChAT is diffusely localized throughout the yeast cytosol (Supplementary Figure 4C). Lastly, by anti-ChAT immunoblotting we observed that ChAT is stably expressed in transformed yeast after 10 h of galactose induction (Supplementary Figure 4D).

**FIGURE 6:**
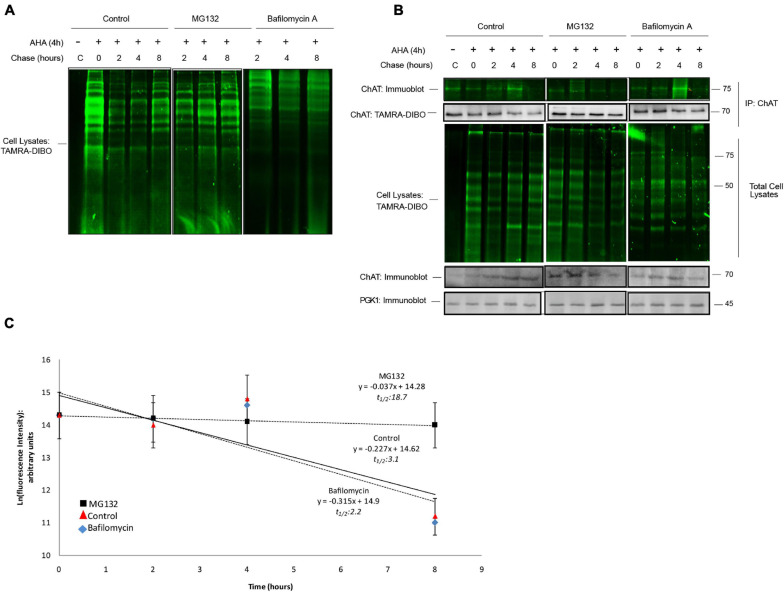
SPAAC pulse-chase analysis of ChAT protein half-life in yeast. **(A)** Fluorescence detection of total AHA-labeled proteins from BY Δ *pdr5* yeast cells following SPAAC pulse-chase (24 h AHA pulse, 0–8 h methionine chase) and labeling with the strained cyclooctyne TAMRA-DIBO. Cells were co-treated with either 50 μM MG132 or 10 μM Bafilomycin A throughout both the 24 h AHA-pulse and 8 h chase periods to inhibit the proteasome or lysosome, respectively. Control unlabeled cells were incubated in media with methionine (i.e., without AHA). **(B)** Fluorescence detection of immunoprecipitated (IP) AHA/TAMRA-labeled human ChAT protein from ChAT-expressing BY Δ *pdr5* yeast cells following SPAAC pulse-chase with the strained cyclooctyne TAMRA-DIBO. Fluorescent AHA/TAMRA-labeled proteins from either whole cell lysates or anti-ChAT IPs were detected in resolved SDS-PAGE gels at an Ex/Em of 555/580 nm. Anti-ChAT and anti-PGK1 immunoblots were completed on AHA/TAMRA-labeled protein samples for downstream data analysis and as loading controls. **(C)** Proteasome inhibition by MG132 treatment increased the protein half-life of human ChAT (18.7 h) as compared to DMSO-treated control yeast cells [3.1 h; *F*(1,46) = 5.6432, *p* ≤ 0.001]. Bafilomycin A co-treatment had no significant effect on the half-life of AHA/TAMRA-labeled ChAT (2.2 h). ChAT fluorescence intensities from **(B)** were plotted on a semi-logarithmic scale and linear regression was completed to determine human ChAT protein half-life (*n* = 5).

We next applied the SPAAC pulse-chase method to ChAT-expressing yeast cells treated with the proteasome inhibitor MG132 (50 μM) or Bafilomycin A (10 μM), an autophagy inhibitor (Yoshimori et al., 1991), throughout both the 24 h AHA-pulse and 8 h chase periods. Similar to mammalian cells, we detected the global progressive loss of fluorescent AHA/TAMRA-labeled proteins from whole cell lysates during the 0–8 h chase period in the absence of changes in total protein levels as measured in parallel by anti-PGK1 immunoblotting as a loading control (Figures 6A,B). Co-treatment of cells with either MG132 or Bafilomycin A reduced the loss of AHA-labeled proteins from whole cell lysates. Importantly, following anti-ChAT IPs we observed progressive loss of fluorescent AHA/TAMRA-labeled ChAT protein during the 0–8 h chase period (Figure 6B). By quantifying the fluorescence intensities of AHA/TAMRA-labeled ChAT (Figure 6C) we determined that the protein half-life of human ChAT is 3.1 h in yeast. Furthermore, we observed that proteasome inhibition by MG132 co-treatment reduced the decay of ChAT protein as compared to DMSO-treated control cells [half-life = 18.7 h; *F*(1,46) = 5.6432, *p* ≤ 0.001; Figure 6C], whereas Bafilomycin A co-treatment had no significant effect on the half-life of AHA/TAMRA-labeled ChAT (2.2 h). It is important to note that live-labeling of yeast cells with AHA did not result in any growth defect regardless of expression of human ChAT (Supplementary Figure 4B).

Lastly, we analyzed the effect of AHA on the HSR in yeast. To accomplish this, we first utilized a fluorescent reporter system (Supplementary Figure 5A) whereby expression of heterologous GFP is driven by binding of heat shock factor 1 (Hsf1) to a synthetic promoter containing four adjacent heat shock elements (HSE) (Sorger et al., 1987) in cells following exposure to hyperthermia or protein misfolding stress (Brandman et al., 2012). Following treatment of yeast cell with AHA for 4 h at 24°C, we observed an increase in GFP-positive cells (Supplementary Figure 5B; *p* ≤ 0.001). As a positive control, we also observed an increase in GFP-positive cells following exposure to hyperthermic stress at 42°C for 1 h (*p* ≤ 0.001), and that exposure of AHA-treated cells to 42°C led to a further two-fold increase in GFP positive cells as compared to hyperthermia alone (*p* ≤ 0.001). Additionally, by immunoblotting (Supplementary Figure 5C) we show that treatment of yeast cells with AHA for 4 h at 24°C increased the steady-state protein levels of endogenous Hsp70, Hsp42, and Hsp104 (*p* ≤ 0.001), while no changes were observed for HSP26 (Supplementary Figure 5D). Lastly, exposure of yeast cells to 42°C for 1 h led to an increase in these HSPs (*p* ≤ 0.001) as compared to AHA-treated cells grown at 24°C. Taken together, these results demonstrate that our SPAAC pulse-chase method can be used successfully in yeast and, while AHA treatment did induce the heat shock response in yeast similar to some mammalian cells, this remained non-toxic and did not affect yeast viability.

## Discussion

Multiple methods are available to cell biologist to study the rate at which cellular proteins are degraded, but each of these approaches have severe limitations and experimental problems (Table 1). Here, we establish a non-toxic and non-radioactive pulse-chase method for the determination of cellular protein half-life that utilizes SPAAC click chemistry reactions. We provide proof-of-principal examples for this method in multiple mammalian cells lines and in yeast using both heterologously expressed (wild-type and mutant human ChAT) and endogenous (tumor suppressor p53) proteins. Furthermore, by applying different commercially available fluorescent and biotin cyclooctyne probes, we demonstrate the versatility and flexibility of this novel method.

**TABLE 1:** Comparison of SPAAC pulse-chase to existing protein half-life methods.

Technique	Advantages	Limitations	References
Cycloheximide (CHX)	• Less time consuming • No need for immunoprecipitation • Minimal cost • Immunoblot detection	• Highly cytotoxic • Off-target effects can alter protein degradation • Typically only usable for short-lived (<6 h) proteins due to toxic effects	Hanna et al., 2003; Schneider-Poetsch et al., 2010; Dai et al., 2013
^35^S-methionine pulse-chase	• Limited cytotoxicity • Gold standard • Can be used for both short- and long-lived proteins	• Radioactive • Special considerations for personnel safety, equipment, and permits • Requires stringent immunoprecipitation • Costly • More time consuming than CHX assays • Can induce DNA damage	Hu and Heikka, 2000; Hu et al., 2001; Esposito and Kinzy, 2014; Takahashi and Ono, 2003
GFP fusion proteins	• Live-cell microscopy • Photoactivatable • Photobleachable	• Requires heterologous expression of fusion proteins • Can induce proteome changes and induce cellular toxicity	Baens et al., 2006; Zhang et al., 2007; Eden et al., 2011; Coumans et al., 2014; Ansari et al., 2016
Strain-promoted alkyne-azide cycloaddition (SPAAC) pulse-chase	• Non-radioactive • Non-toxic • No effect on global ubiquitination • Fluorescent or chemiluminescent • Adaptable for end-user equipment and cell models • Can be used for both short- and long-lived proteins	• Requires stringent immunoprecipitation • More time consuming than CHX assays • Can induce the heat shock response	Dieterich et al., 2006, 2010; Baskin et al., 2007; Roche et al., 2009; McShane et al., 2016; Morey et al., 2016

The bioorthogonal amino acid AHA is a non-toxic methionine analog that can incorporate into newly synthesized proteins without altering global rates of protein degradation or ubiquitination (Kiick et al., 2002; Dieterich et al., 2006). AHA has been used widely in both *in vitro* and *in vivo* studies to measure global changes in protein degradation (Kiick et al., 2002; Dieterich et al., 2006, 2010; Baskin et al., 2007; Roche et al., 2009; McShane et al., 2016) and is available commercially or can be synthesized in-house (Link et al., 2007; Roth et al., 2010). While considered a non-obtrusive replacement for methionine in pulse-chase studies (Ma and Yates, 2018; Steward et al., 2020), one potential caveat of using AHA is that its incorporation into nascent proteins may alter protein folding, thus leading to induction of the heat shock and/or misfolded protein response. Our results indicate that AHA labeling can induce the HSR in both mammalian and yeast cells. It is important to note, however, that in mammalian cells this induction was significantly less than that observed following treatment with AZC, a proline analog known to induce protein misfolding (Fowden and Richmond, 1967; Trotter et al., 2001), or in yeast cells exposed to hyperthermic conditions. Additionally, AHA incorporation did not induce the HSR in human HEK293 cells, suggesting that this effect of AHA is not universal. Importantly, in support of the growing literature on the cellular safety of AHA, we provide further evidence that, in mammalian cells, AHA does not induce apoptosis nor lead to changes in global ubiquitination, does not promote accumulation of insoluble ubiquitinated proteins, and does not affect yeast cell growth. Furthermore, separate studies have used AHA to label proteins in live animals without negatively affecting animal behavior, growth and development, and physiology (Hinz et al., 2012; Calve et al., 2016). Lastly, Lehner et al. (2017) reported that in *in vitro* studies AHA labeling of recombinant PDZ3 domain proteins results in only minor alterations to protein secondary structure while not affecting ligand binding and yielded a soluble, well-folded, and functional model protein. Thus, while AHA incorporation could introduce changes to protein folding, these effects appear to be minor and/or negligible, and our work together with the aforementioned studies suggests that AHA is a non-toxic methionine analog that is minimally invasive to cell physiology and is suitable for use in pulse-chase studies.

One limitation of SPAAC pulse-chase is that labeling of nascent proteins with AHA is directly proportional to the number of methionine residues in a given protein. Thus, if a protein-of-interest contains a small number of methionine molecules, or only contains the N-terminal methionine that is often excised during post-translational processing (Giglione et al., 2004), detection of nascent proteins with AHA may be difficult. Fortunately, additional azide-containing bioorthogonal amino acids are available for labeling either phenylalanine (4-azido-L-phenylalanine) or tyrosine (4-propargyloxy-L-phenylalanine) residues, though use of these require specialized engineered cells expressing tRNAs that can accept these bioorthogonal amino acids (Saleh et al., 2019). An alternative strategy to AHA involves the labeling of newly synthesized proteins in the presence of methionine with O-propargyl-puromycin (OPP), an alkyne-containing puromycin analog that forms covalent linkages with the C-terminus of nascent polypeptides (Liu et al., 2012; Forester et al., 2018; Hidalgo San Jose and Signer, 2019). It is important to note though that incorporation of OPP into nascent proteins results in premature translation termination, release of C-terminally truncated peptides from the ribosome, and an overall inhibition of protein synthesis similar to that observed with CHX (Liu et al., 2012; Forester et al., 2018).

One of the unique advantages to SPAAC pulse-chase is the ever-growing selection of commercially available reagents for SPAAC reactions, including various bioorthogonal amino acids as discussed above and complementary fluorescent or chemiluminescent probes. This offers a large degree of flexibility in experimental design and increased compatibility with commonly used SDS-PAGE and immunoblotting equipment. To demonstrate the flexibility of SPAAC pulse-chase, we used three different cyclooctyne probes, including fluorescent TAMRA-DIBO or 488-DBCO, or a biotinylated DIBO compound detected by chemiluminescence using streptavidin-HRP. Importantly, we observed little difference in the sensitivities of these probes and obtained similar results when measuring the protein half-life of endogenous p53, suggesting that these and potentially other cyclooctyne probes may be interchangeable. Taken together, by using click chemistry reagents this SPAAC pulse-chase method is modular and adaptable to a variety of experimental needs, limitations, and available resources.

In conclusion, we present a novel, non-toxic, and non-radioactive method as an alternative to classical ^35^S-methionine live-labeling or CHX experiments for the determination of protein half-life. Importantly, this method utilizes bioorthogonal click chemistry reactions in a manner that is both compatible with different eukaryotic systems and that allows for end-user customization. We believe that this protocol will be of interest to and applicable to many researchers in the fields of molecular and cellular biology. Lastly, this method is an important example of the potential for click chemistry to improve existing methods and of the growing utility of click chemistry in studying biological systems.

## Data Availability Statement

The raw data supporting the conclusions of this article will be made available by the authors, without undue reservation.

## Author Contributions

TMM, MAE, MLD, and RJR designed the experiments. TMM completed experimental work detailing SPAAC pulse-chase in mammalian cells, while MAE performed experiments in yeast. All authors assisted with data analysis and contributed to writing this manuscript through editing and revisions.

## Conflict of Interest

The authors declare that the research was conducted in the absence of any commercial or financial relationships that could be construed as a potential conflict of interest.

## Publisher’s Note

All claims expressed in this article are solely those of the authors and do not necessarily represent those of their affiliated organizations, or those of the publisher, the editors and the reviewers. Any product that may be evaluated in this article, or claim that may be made by its manufacturer, is not guaranteed or endorsed by the publisher.

## Footnotes

1
http://cshprotocols.cshlp.org/site/recipes/nav_y.dtl

## Supplementary Material

Supplementary Figure 1 Analysis of cell viability, global proteome ubiquitination, and heat shock response in mouse cholinergic SN56 cells labeled with AHA. **(A)** SN56 cells were live-labeled in culture for 4 h with 50 μM AHA and either collected immediately (0 h) or following 8 h of chase in methionine-containing media. Control unlabeled cells were incubated in media with methionine (i.e., without AHA). Cells were lysed and AHA-labeled protein samples from whole cell lysates were reacted with the strained cyclooctyne TAMRA-DIBO. Protein samples were run on SDS-PAGE gels, AHA-labeled proteins were detected in-gel at an Ex/Em 555/580 nm, samples were transferred to PVDF membranes, and immunoblotting was completed as indicated. In support of previous studies (Dieterich et al., 2006), incorporation of AHA into newly synthesized proteins was prevented when cells were co-treated with CHX, a potent inhibitor of protein synthesis. Additionally, incubation of cells with AHA alone failed to induce apoptosis **(B)**, nor did this lead to changes in total protein ubiquitination **(C)** as compared to unlabeled cells. Conversely, treatment of cells with 100 μg/ml of cycloheximide (CHX) throughout both the 4 h AHA pulse and the 8 h chase periods (up-to 12 h total) both induced apoptosis (**B**; ^∗^*p* ≤ 0.05) and led to a depletion of total ubiquitinated proteins (**C**; ^∗∗^*p* ≤ 0.01) as compared to control untreated cells. As positive controls, treatment of unlabeled cells with 200 nM staurosporine, a pan-kinase inhibitor (Karaman et al., 2008), for 8 h induced apoptosis (**B**; ^∗∗∗^*p* ≤ 0.01) while treatment with 10 mM azetidine-2-caroxylic acid (AZC), a proline analog that can induce protein misfolding (Weids et al., 2016), for 24 h led to the enhanced accumulation of total ubiquitinated proteins (**C**; ^∗∗∗^*p* ≤ 0.001) as compared to AHA-labeled cells. Induction of cellular apoptosis was measured by immunoblotting for the formation of a lower molecular mass cleaved form of poly (ADP-ribose) polymerase (PARP) (Mullen, 2004), while levels of total ubiquitinated proteins was measured by anti-ubiquitin immunoblotting. To measure induction of the heat shock response following cell labeling with AHA, immunoblotting for the heat shock proteins HSP70, HSP90, and HSC70 was completed. Interestingly, enhanced synthesis of HSP70 **(A)**, HSP90 (**D**; ^∗^*p* ≤ 0.05), and HSC70 (**E**; *p* = 0.09) was observed in cells incubated with AHA and chased for 8 h as compared to control untreated cells; this was prevented when AHA-labeled cells were co-treated with CHX. Furthermore, as a positive control treatment of unlabeled cells with 10 mM AZC for 24 h promoted the synthesis of HSP70 **(A)**, HSP90 (**D**; ^∗∗∗^*p* ≤ 0.01), and HSC70 (**E**; ^∗∗∗^*p* ≤ 0.01). The induction of HSP70 expression was greater in cells treated with AZC as compared to cells labeled with AHA (**F**; ^∗∗∗^*p* ≤ 0.01). Statistical analysis was completed by one-way ANOVA with Dunnett’s *post hoc* test (mean ± SEM, *n* = 5).

Supplementary Figure 2 Incubation of cells with AHA does not affect overall solubility of cellular proteins. **(A)** SN56 cells were live-labeled in culture for 4 h with 50 μM AHA and either collected immediately (0 h) or following 8 h of chase in methionine-containing media. Control unlabeled cells were incubated in media with methionine (i.e., without AHA). Cells were lysed in buffer containing 0.1% Triton X-100 and fractionated into soluble and insoluble proteins. Triton X-100 insoluble proteins were solubilized prior to SDS-PAGE by denaturing in 2× Laemmli sample buffer with 5% 2-mercaptoethanol. Following transfer to PVDF membranes, anti-ubiquitin immunoblotting was completed to detect levels of ubiquitinated cellular proteins in Triton-soluble and Triton-insoluble fractions. While incubation of cells with AHA led to the synthesis of the heat shock protein HSP70 as observed in Supplementary Figure 1, this was only observable in the Triton-soluble fraction. Furthermore, there was no detectable change in the abundance of total ubiquitinated proteins following incubation with AHA in either the Triton-soluble **(B)** or Triton–insoluble fraction **(C)** as compared to unlabeled cells. As a positive control to induce protein misfolding, treatment of unlabeled cells with 10 mM AZC for 24 h promoted the synthesis of HSP70 (observable in both fractions) and led to the enhanced accumulation of total ubiquitinated proteins in both the Triton-soluble (**B**; ^∗∗∗^*p* ≤ 0.001) and Triton–insoluble fraction (**C**; ^∗∗∗^*p* ≤ 0.001) as compared to AHA-treated cells. Statistical analysis was completed by one-way ANOVA with Tukey’s *post hoc* test (mean ± SEM, *n* = 3).

Supplementary Figure 3 Analysis of cell viability, global proteome ubiquitination, and heat shock response in human HEK293 and HeLa cells labeled with AHA. **(A)** HEK293 or HeLa cells were live-labeled in culture for 4 h with 50 μM AHA and either collected immediately (0 h) or following 8 h of chase in methionine-containing media. Control unlabeled cells were incubated in media with methionine (i.e., without AHA). As a positive control, unlabeled cells were treated with either 10 mM AZC for 24 h to induce protein misfolding. Cells were lysed and AHA-labeled protein samples from whole cell lysates were reacted with the strained cyclooctyne TAMRA-DIBO. Protein samples were run on SDS-PAGE gels, AHA-labeled proteins were detected in-gel at an Ex/Em 555/580 nm, samples were transferred to PVDF membranes, and immunoblotting was completed as indicated. Similar to SN56 cells (Supplementary Figure 1), incubation with AHA failed to induce apoptosis (**B** – HEK293; **C** – HeLa) or lead to changes in total protein ubiquitination in either HEK293 **(D)** or HeLa cells **(E)** as compared to unlabeled cells. Conversely, treatment of cells with AZC induced apoptosis (**B** – HEK293; **C** – HeLa; ^∗∗∗^*p* ≤ 0.001) and enhanced the accumulation of total ubiquitinated proteins in either HEK293 (**D**; ^∗∗∗^*p* ≤ 0.001) or HeLa cells (**E**; ^∗∗∗^*p* ≤ 0.001) as compared to AHA-labeled cells. Induction of cellular apoptosis was measured by immunoblotting for the formation of a lower molecular mass cleaved form of PARP, while levels of total ubiquitinated proteins was measured by anti-ubiquitin immunoblotting. To measure induction of the heat shock response following cell labeling with AHA, immunoblotting for the heat shock proteins HSP90, HSP70, and HSC70 was completed. In HEK293 cells, AHA labeling had no effect on the steady-state levels of HSP90 **(F)**, HSP70 **(H)**, or HSC70 **(J)**, while treatment with AZC did lead to increased protein levels of HSP90 (**F**; ^∗∗^*p* ≤ 0.01) as compared to unlabeled and AHA-treated cells. In HeLa cells, labeling of cells with AHA and chasing them for 8 h led to increased steady-state protein levels of HSP90 (**G**; ^∗^*p* ≤ 0.05) and HSP70 (**I**; ^∗∗∗^*p* ≤ 0.001) as compared to control unlabeled cells; HSC70 protein levels were unchanged in HeLa cells following AHA labeling **(K)**. Lastly, treatment of HeLa cells with AZC increased the protein levels of HSP90 (**G**; ^∗∗∗^*p* ≤ 0.001), HSP70 (**I**; ^∗∗∗^*p* ≤ 0.001), and HSC70 (**K**; *p* = 0.06) as compared to control unlabeled cells. Statistical analysis was completed by one-way ANOVA with Tukey’s *post hoc* test (mean ± SEM, *n* = 4).

Supplementary Figure 4 AHA labeling in yeast cells is non-toxic. **(A)** Growth assays on plates of yeast cells expressing ChAT and controls. Liquid cultures of yeast were spotting on selective inducing agar media and incubated at 30°C for 2–4 days before imaging. **(B)** Growth curves of Δpdr5 yeast cells expressing ChAT and controls grown in the presence or absence of 50 μM L-azidohomoalanine (AHA). **(C)** Fluorescence microcopy of yeast expressing ChAT-YFP. Cells were imaged using a Leica TCS SP5 II confocal microscope at 63× magnification. The scale bar represents 5 μm. **(D)** Immunoblots of protein lysates of yeast cells expressing ChAT and controls grown in the presence of AHA probed with anti-ChAT or anti-histone H3 antibodies.

Supplementary Figure 5 AHA labeling induces a mild heat shock response in yeast. **(A)** Fluorescence microscopy of yeast cells expressing HSE-eGFP grown for 4 h in the presence or absence of AHA at 24 and 42°C and quantification (**B**; ^∗^*p* ≤ 0.05, ^∗∗^*p* ≤ 0.01; *n* = 5). **(C)** Anti-HSP immunoblots prepared with protein lysates from yeast cells grown in the presence or absence of AHA at 24 or 42°C and quantification **(D)**. Anti-histone H3 immunoblotting was completed as a loading control.

## Abbreviations

ACh: acetylcholine
AHA: L-azidohomoalanine
AZC: azetidine-2-caroxylic acid
ChAT: choline acetyltransferase
CHX: cycloheximide
CMS: congenital myasthenic syndrome
CTab: anti-ChAT carboxyl-terminal peptide antibody
DBCO: dibenzocyclooctyne
DIBO: 4-dibenzocyclooctynol
GFP: green fluorescent protein
HSC70: heat shock cognate 71 kDa protein
HSE: heat shock element
HSF1: heat shock factor 1
HSP: heat shock protein
HSR: heat shock response
IP: immunoprecipitation
NEM: N-ethylmaleimide
OPP: O-propargyl -puromycin
p53: cellular tumor antigen p53
PARP: poly (ADP-ribose) polymerase
PGK1: phosphoglycerate kinase 1
PVDF: polyvinylidene fluoride
RIPA: radioimmune precipitation buffer
SDS-PAGE: sodium dodecyl sulfate polyacrylamide gel electrophoresis
SPAAC: strain-promoted alkyne-azide cycloaddition
TAMRA: tetramethylrhodamine
WT: wild-type
YFP: yellow fluorescent protein.
