# Supplementary figures and images for: SPAAC Pulse-Chase: A Novel Click Chemistry-Based Method to Determine the Half-Life of Cellular Proteins
Source: Front Cell Dev Biol. 2021 Sep 7;9:722560. doi: 10.3389/fcell.2021.722560 (PMC8452969; doi:10.3389/fcell.2021.722560)

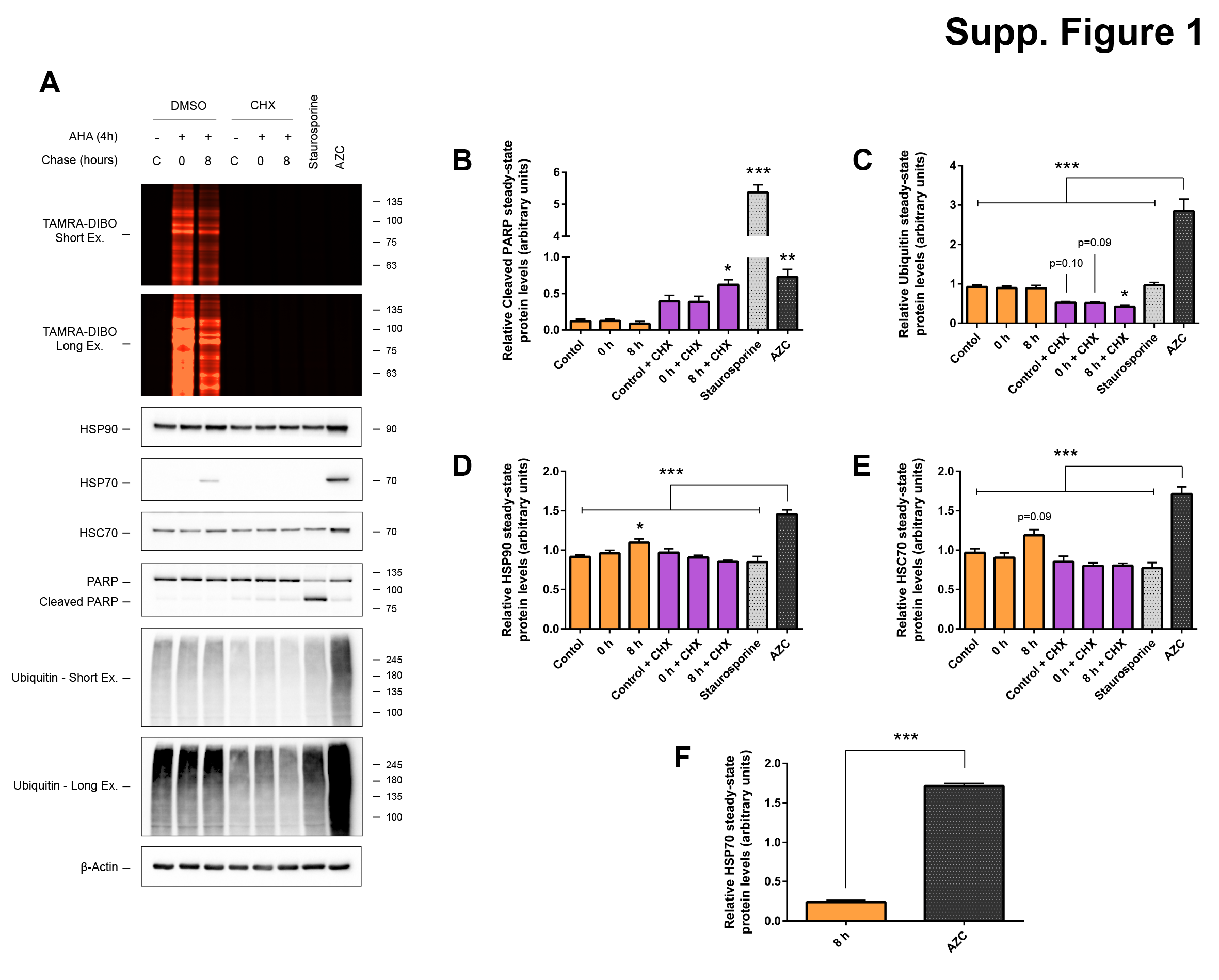

Supplement: Supplementary Figure 1 — Analysis of cell viability, global proteome ubiquitination, and heat shock response in mouse cholinergic SN56 cells labeled with AHA. (A) SN56 cells were live-labeled in culture for 4 h with 50 μM AHA and either collected immediately (0 h) or following 8 h of chase in methionine-containing media. Control unlabeled cells were incubated in media with methionine (i.e., without AHA). Cells were lysed and AHA-labeled protein samples from whole cell lysates were reacted with the strained cyclooctyne TAMRA-DIBO. Protein samples were run on SDS-PAGE gels, AHA-labeled proteins were detected in-gel at an Ex/Em 555/580 nm, samples were transferred to PVDF membranes, and immunoblotting was completed as indicated. In support of previous studies (Dieterich et al., 2006), incorporation of AHA into newly synthesized proteins was prevented when cells were co-treated with CHX, a potent inhibitor of protein synthesis. Additionally, incubation of cells with AHA alone failed to induce apoptosis (B), nor did this lead to changes in total protein ubiquitination (C) as compared to unlabeled cells. Conversely, treatment of cells with 100 μg/ml of cycloheximide (CHX) throughout both the 4 h AHA pulse and the 8 h chase periods (up-to 12 h total) both induced apoptosis (B; ∗p ≤ 0.05) and led to a depletion of total ubiquitinated proteins (C; ∗∗p ≤ 0.01) as compared to control untreated cells. As positive controls, treatment of unlabeled cells with 200 nM staurosporine, a pan-kinase inhibitor (Karaman et al., 2008), for 8 h induced apoptosis (B; ∗∗∗p ≤ 0.01) while treatment with 10 mM azetidine-2-caroxylic acid (AZC), a proline analog that can induce protein misfolding (Weids et al., 2016), for 24 h led to the enhanced accumulation of total ubiquitinated proteins (C; ∗∗∗p ≤ 0.001) as compared to AHA-labeled cells. Induction of cellular apoptosis was measured by immunoblotting for the formation of a lower molecular mass cleaved form of poly (ADP-ribose) polymerase (PARP) (Mullen, 2004), while le [file Image_1.TIF]

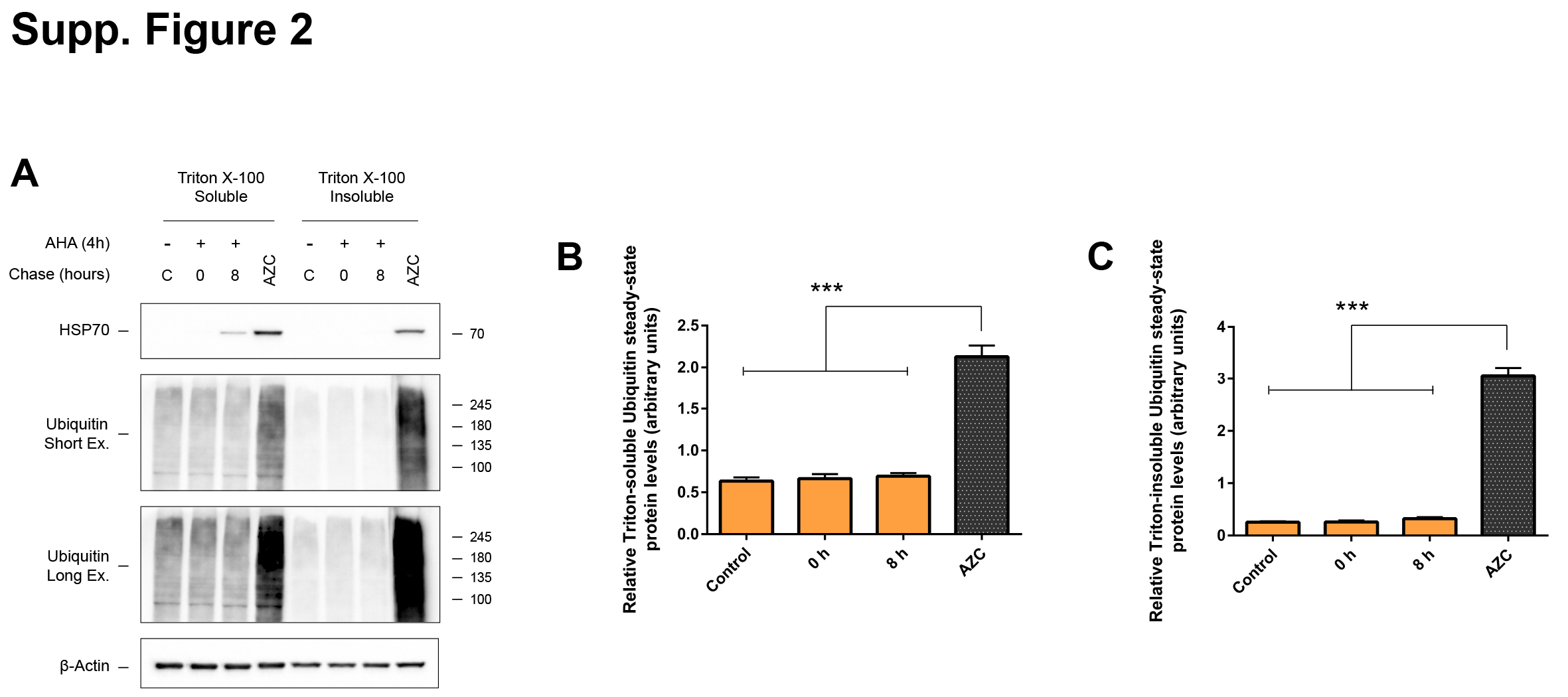

Supplement: Supplementary Figure 2 — Incubation of cells with AHA does not affect overall solubility of cellular proteins. (A) SN56 cells were live-labeled in culture for 4 h with 50 μM AHA and either collected immediately (0 h) or following 8 h of chase in methionine-containing media. Control unlabeled cells were incubated in media with methionine (i.e., without AHA). Cells were lysed in buffer containing 0.1% Triton X-100 and fractionated into soluble and insoluble proteins. Triton X-100 insoluble proteins were solubilized prior to SDS-PAGE by denaturing in 2× Laemmli sample buffer with 5% 2-mercaptoethanol. Following transfer to PVDF membranes, anti-ubiquitin immunoblotting was completed to detect levels of ubiquitinated cellular proteins in Triton-soluble and Triton-insoluble fractions. While incubation of cells with AHA led to the synthesis of the heat shock protein HSP70 as observed in Supplementary Figure 1, this was only observable in the Triton-soluble fraction. Furthermore, there was no detectable change in the abundance of total ubiquitinated proteins following incubation with AHA in either the Triton-soluble (B) or Triton–insoluble fraction (C) as compared to unlabeled cells. As a positive control to induce protein misfolding, treatment of unlabeled cells with 10 mM AZC for 24 h promoted the synthesis of HSP70 (observable in both fractions) and led to the enhanced accumulation of total ubiquitinated proteins in both the Triton-soluble (B; ∗∗∗p ≤ 0.001) and Triton–insoluble fraction (C; ∗∗∗p ≤ 0.001) as compared to AHA-treated cells. Statistical analysis was completed by one-way ANOVA with Tukey’s post hoc test (mean ± SEM, n = 3). [file Image_2.tif]

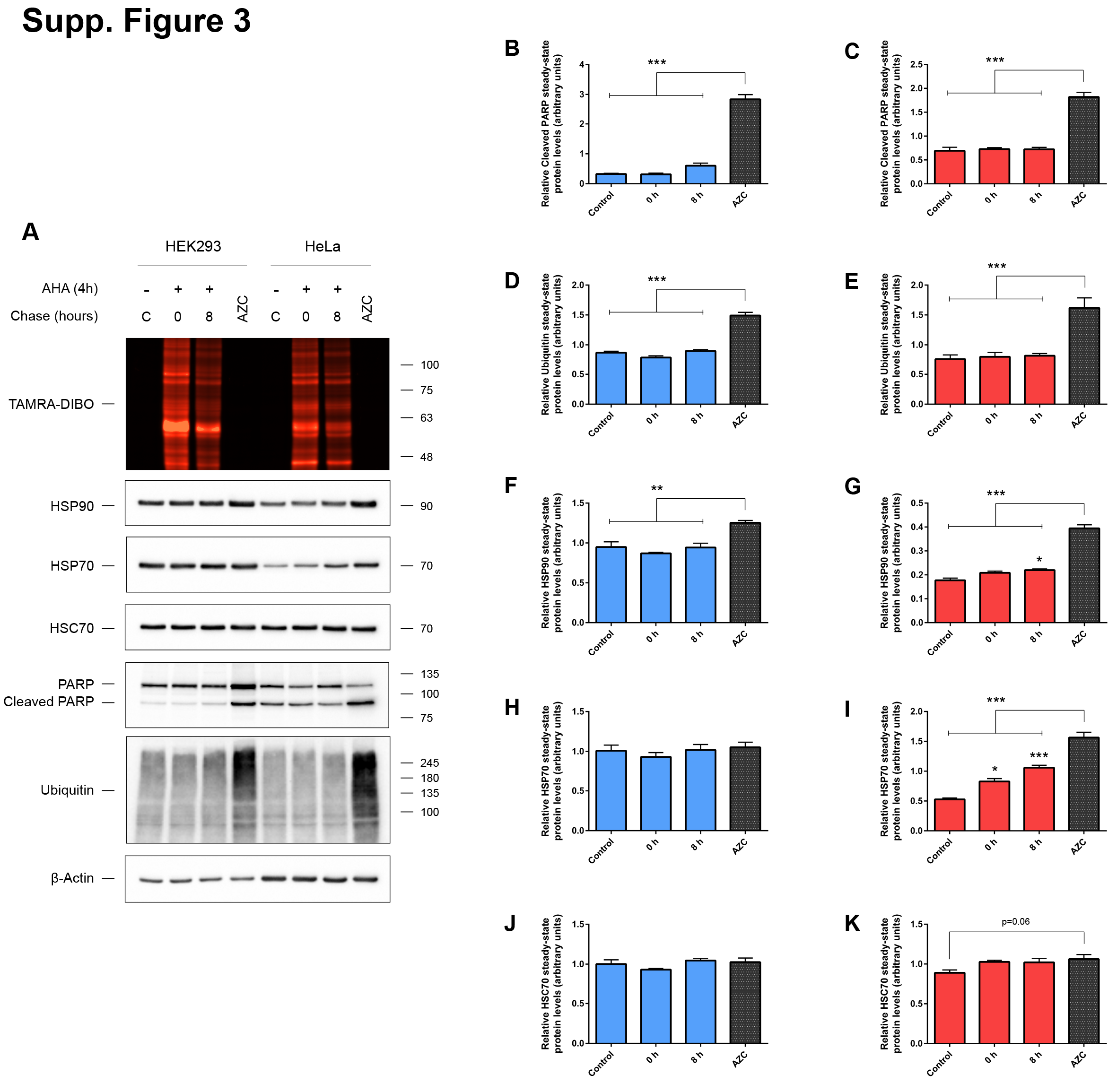

Supplement: Supplementary Figure 3 — Analysis of cell viability, global proteome ubiquitination, and heat shock response in human HEK293 and HeLa cells labeled with AHA. (A) HEK293 or HeLa cells were live-labeled in culture for 4 h with 50 μM AHA and either collected immediately (0 h) or following 8 h of chase in methionine-containing media. Control unlabeled cells were incubated in media with methionine (i.e., without AHA). As a positive control, unlabeled cells were treated with either 10 mM AZC for 24 h to induce protein misfolding. Cells were lysed and AHA-labeled protein samples from whole cell lysates were reacted with the strained cyclooctyne TAMRA-DIBO. Protein samples were run on SDS-PAGE gels, AHA-labeled proteins were detected in-gel at an Ex/Em 555/580 nm, samples were transferred to PVDF membranes, and immunoblotting was completed as indicated. Similar to SN56 cells (Supplementary Figure 1), incubation with AHA failed to induce apoptosis (B – HEK293; C – HeLa) or lead to changes in total protein ubiquitination in either HEK293 (D) or HeLa cells (E) as compared to unlabeled cells. Conversely, treatment of cells with AZC induced apoptosis (B – HEK293; C – HeLa; ∗∗∗p ≤ 0.001) and enhanced the accumulation of total ubiquitinated proteins in either HEK293 (D; ∗∗∗p ≤ 0.001) or HeLa cells (E; ∗∗∗p ≤ 0.001) as compared to AHA-labeled cells. Induction of cellular apoptosis was measured by immunoblotting for the formation of a lower molecular mass cleaved form of PARP, while levels of total ubiquitinated proteins was measured by anti-ubiquitin immunoblotting. To measure induction of the heat shock response following cell labeling with AHA, immunoblotting for the heat shock proteins HSP90, HSP70, and HSC70 was completed. In HEK293 cells, AHA labeling had no effect on the steady-state levels of HSP90 (F), HSP70 (H), or HSC70 (J), while treatment with AZC did lead to increased protein levels of HSP90 (F; ∗∗p ≤ 0.01) as compared to unlabeled and AHA-treated cells. In HeLa cells, labeling of cells with A [file Image_3.TIF]

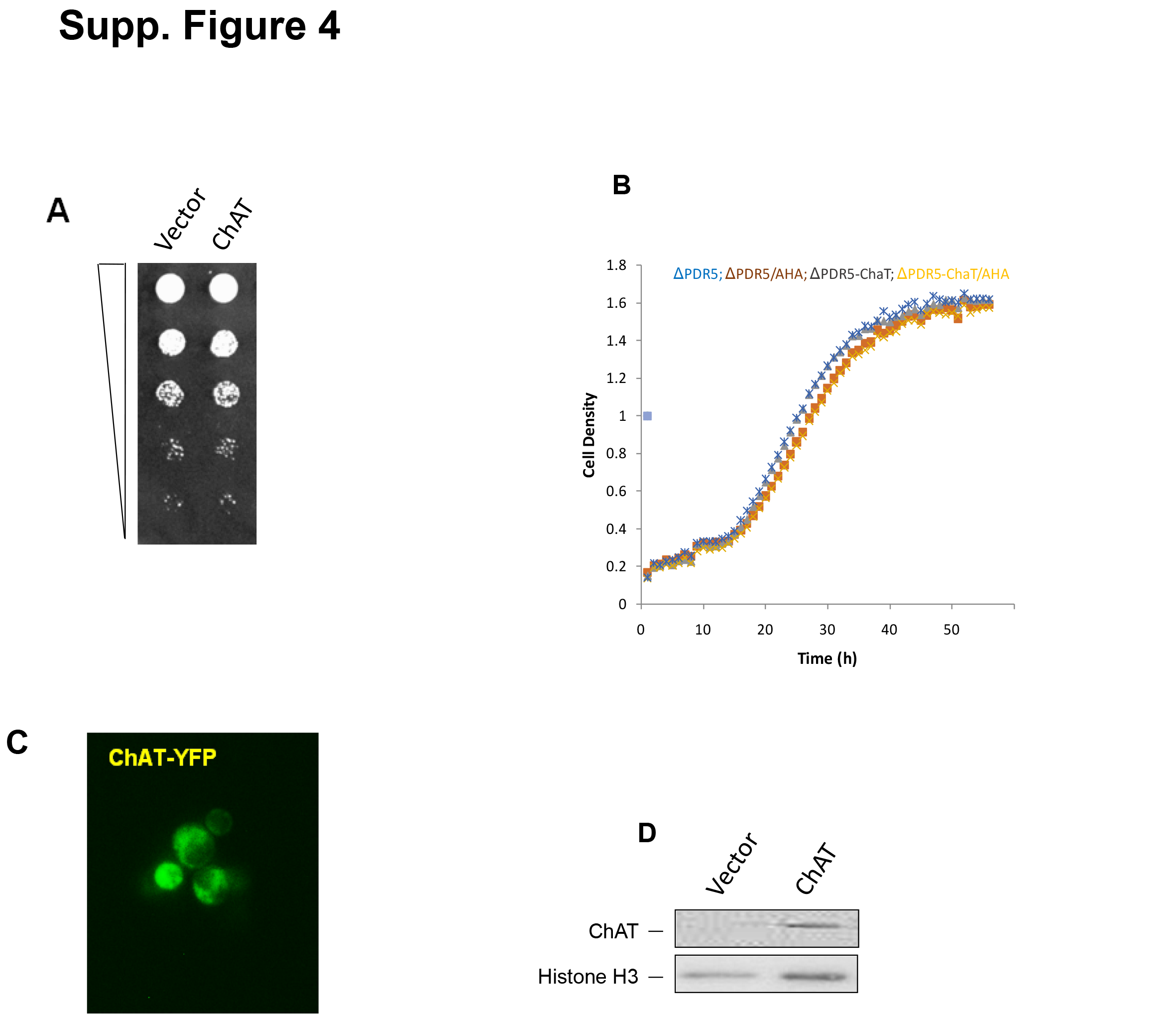

Supplement: Supplementary Figure 4 — AHA labeling in yeast cells is non-toxic. (A) Growth assays on plates of yeast cells expressing ChAT and controls. Liquid cultures of yeast were spotting on selective inducing agar media and incubated at 30°C for 2–4 days before imaging. (B) Growth curves of Δpdr5 yeast cells expressing ChAT and controls grown in the presence or absence of 50 μM L-azidohomoalanine (AHA). (C) Fluorescence microcopy of yeast expressing ChAT-YFP. Cells were imaged using a Leica TCS SP5 II confocal microscope at 63× magnification. The scale bar represents 5 μm. (D) Immunoblots of protein lysates of yeast cells expressing ChAT and controls grown in the presence of AHA probed with anti-ChAT or anti-histone H3 antibodies. [file Image_4.TIF]

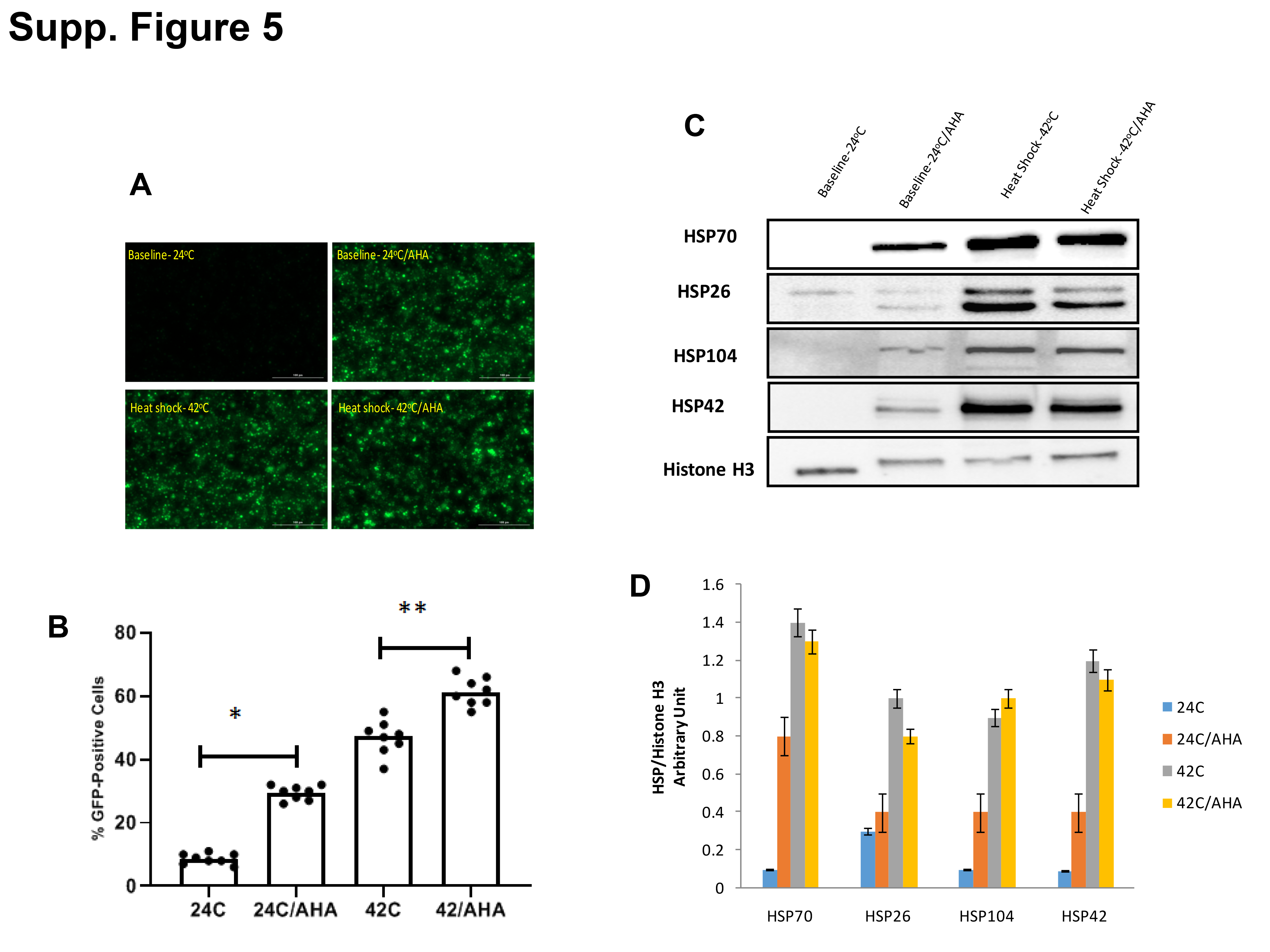

Supplement: Supplementary Figure 5 — AHA labeling induces a mild heat shock response in yeast. (A) Fluorescence microscopy of yeast cells expressing HSE-eGFP grown for 4 h in the presence or absence of AHA at 24 and 42°C and quantification (B; ∗p ≤ 0.05, ∗∗p ≤ 0.01; n = 5). (C) Anti-HSP immunoblots prepared with protein lysates from yeast cells grown in the presence or absence of AHA at 24 or 42°C and quantification (D). Anti-histone H3 immunoblotting was completed as a loading control. [file Image_5.tif]
